# Multiomics unravels the complexity of male obesity: a prospective observational study

**DOI:** 10.1186/s12967-024-06040-7

**Published:** 2025-01-30

**Authors:** Georgios E. Papadakis, Lucie Favre, Yassine Zouaghi, Nathalie Vionnet, Nicolas J. Niederländer, Michela Adamo, James S. Acierno, Dassine Berdous, Alexia Boizot, Jenny Meylan, Julijana Ivanisevic, Emmanuelle Paccou, Hector Gallart-Ayala, Tim Reyns, Elise Van Caeneghem, Bruno Lapauw, Jérôme Pasquier, Yasser Aleman, Styliani Mantziari, Olivier Salamin, Raul Nicoli, Tiia Kuuranne, Tom Fiers, Patric Hagmann, Federico Santoni, Andrea Messina, Nelly Pitteloud

**Affiliations:** 1 Department of Endocrinology, Diabetology and Metabolism, Lausanne University Hospital, Avenue de la Sallaz 8, CH-1011 Lausanne, Switzerland; 2 Faculty of Biology and Medicine, University of Lausanne, Rue du Bugnon 21, CH-1005 Lausanne, Switzerland; 3 Metabolomics Platform, Faculty of Biology and Medicine, University of Lausanne, Rue du Bugnon 19, CH-1005 Lausanne, Switzerland; 4 Department of Clinical Chemistry, Ghent University Hospital, 9000 Ghent, Belgium; 5 Center for Primary Care and Public Health, University of Lausanne, CH-1011 Lausanne, Switzerland; 6 Division of Radio-Diagnostics and Interventional Radiology, Lausanne University Hospital, Rue du Bugnon 46, CH-1011 Lausanne, Switzerland; 7 Department of Visceral Surgery, Lausanne University Hospital, Rue du Bugnon 46, CH-1011 Lausanne, Switzerland; 8 Swiss Laboratory for Doping Analyses, University Center of Legal Medicine, Lausanne University Hospital and University of Geneva, Chemin de La Vulliette 4, CH-1000 Lausanne, Switzerland

**Keywords:** Male obesity, Metabolic risk stratification, Hypogonadism, Bariatric surgery, Transcriptomics

## Abstract

**Background:**

Obesity is associated with varying degrees of metabolic dysfunction. In this study, we aimed to discover markers of the severity of metabolic impairment in men with obesity via a multiomics approach.

**Methods:**

Thirty-two morbidly men with obesity who were candidates for Roux-en-Y gastric bypass (RYGB) surgery were prospectively followed. Nine healthy adults served as controls. Deep phenotyping, including targeted metabolomics, transcriptomics, and brain magnetic resonance imaging (MRI), was performed.

**Results:**

Testosterone emerged as a key contributor to phenotypic variability via principal component analysis and was therefore used to further categorize obese patients as having or not having hypogonadotropic hypogonadism (HH). Despite having comparable body mass indices, obese individuals with HH presented with worse metabolic defects than obese individuals without HH, including higher insulin resistance, as well as MRI signs of hypothalamic inflammation and a specific blood transcriptomics signature. The upregulated genes were involved mainly in inflammation, mitochondrial function, and protein translation. Integration of gene expression and clinical data revealed high FGF21 and low cortisol levels as the top markers correlated with the transcriptomic signature of metabolic risk. Following RYGB-induced substantial weight loss, testosterone levels markedly increased in both obese individuals with and without HH, challenging the current definition of hypogonadism. A longitudinal study in a subset of men with obesity following bariatric surgery revealed a unique FGF21 trajectory with a sharp peak at one month post-RYGB that correlated with metabolic and reproductive improvements.

**Conclusions:**

Combining clinical, biochemical, and molecular markers allows adequate stratification of metabolic risk in men with obesity and provides novel tools for personalized care.

**Supplementary Information:**

The online version contains supplementary material available at 10.1186/s12967-024-06040-7.

## Background

Obesity is associated with high morbidity, particularly metabolic syndrome (MetS), type 2 diabetes (T2D), and cardiovascular disease [1]. Metabolically healthy obesity, defined as a body mass index (BMI) > 30 kg/m^2^ and no other MetS component, also confers health risks in the medium term [2]. Thus, there is an imperative need to identify novel markers of metabolic dysfunction to better cluster obese populations and identify those who require more intensive management.

Male obesity is often associated with low testosterone (T) and low or inappropriately normal luteinizing hormone (LH) levels, a constellation termed hypogonadotropic hypogonadism (HH) [3]. The reduction in T levels is the consequence of both decreased hepatic production of sex-hormone binding globulin (SHBG) due to insulin resistance and true inhibition of the hypothalamic‒pituitary‒gonadal (HPG) axis [4]. HH can in turn exacerbate metabolic syndrome by removing the metabolic benefits of T (i.e.*, effects* on body composition and insulin sensitivity) [5]. Whether systemic and, more specifically, central inflammation contributes to HPG axis inhibition in male obesity is currently unknown.

Further elucidating the vicious cycle between MetS and hypogonadism is crucial. Among the more recently identified metabolic cues, Fibroblast growth factor-21 (FGF21) has emerged as a critical regulator of energy homeostasis, and its expression increases in the liver upon metabolic stress [6]. Additionally, FGF21 can cross the blood‒brain barrier [7] and act centrally to modulate energy expenditure [8]. Interestingly, both the loss of FGF21 signaling (in mice and humans) and the overexpression of fgf21 (in mice) alter the hypothalamic control of reproduction [9, 10]. Taken together, these data highlight FGF21 as a plausible hormonal link between male obesity and hypogonadism that merits further investigation.

Obesity-induced HH is mostly reversible following significant weight reduction [11]. Bariatric surgery is currently the most effective way to achieve sustained weight loss [12]. The hormonal changes underlying the metabolic and reproductive benefits of RYGB are still under investigation. In particular, previous observations on the post-RYGB FGF21 changes were derived mostly from female cohorts and showed contradicting results [13–16]. Moreover, studies with detailed longitudinal follow-up are lacking.

Herein, we conducted a prospective study on men with obesity awaiting Roux-en-Y gastric bypass (RYGB). Our aims were (i) to perform a complete characterization of the phenotypic spectrum of male obesity to identify novel markers of metabolic dysfunction, (ii) to explore the role of central inflammation in obesity-associated HH, and (iii) to investigate the contribution of FGF21 to the benefits of bariatric surgery.

## Methods

### Setting and study population

This was a prospective observational study conducted at Lausanne University Hospital. The research protocol was approved by the Institutional Ethics Committee for Research of the Canton of Vaud, Switzerland (CER-VD), and written informed consent was obtained. We recruited men with obesity (age 18–60 years, BMI > 35 kg/m^2^) awaiting RYGB between 2016 and 2020 (Fig. S1). Subjects with primary hypogonadism, hyperprolactinemia or severe chronic nonendocrine disorders (i.e., severe cardiomyopathy) were excluded. All patients had normal thyroid function and iron studies. Pituitary MRI to exclude a hypothalamic‒pituitary expansive mass was performed in the case of suspicious symptoms (i.e., headaches) or hypopituitarism.

The sample size calculation was based on our primary objective to identify predictors of metabolic dysfunction in men with obesity. For this purpose, we used the raw data from a prior study assessing the change in serum T levels after an oral glucose tolerance test (OGTT) in 74 men, of whom thirty-five had obesity [17]. Among the latter, men with low T levels (ObHH, T < 10.4 nmol/l = 300 ng/dl) had homeostatic model assessment for insulin resistance (HOMA-IR) values of 5.72 ± 2.29, whereas those with normal T levels (ObnHH) were 3.49 ± 2.15. With a type I error probability (two-tailed α) of 0.05 and a statistical power of 0.80, the inclusion of 32 patients with obesity (using a Welch’s t test) would be sufficient to detect a significant elevation in HOMA-IR in hypogonadal versus eugonadal men with obesity. In addition, nine healthy men with a BMI of 18–25 kg/m^2^ served as lean controls.

### Study design

#### Protocol 1

At baseline, all participants underwent the following phenotypic studies.

(i) **Body composition** by a whole-body dual X-ray absorptiometry scan. Fat mass (FM) and visceral adipose tissue (VAT) were calculated as previously described [18].
(ii) **A standardized 2-h OGTT** was performed with 75 g of glucose. The participants were classified into 3 groups according to the American Diabetes Association guidelines (https://www.diabetes.org/): normal glucose tolerance, impaired glucose tolerance, or type 2 diabetes (T2D). Nine men on antidiabetic medications were classified as having T2D. Metabolic syndrome was defined on the basis of the ATP-III criteria [19]. Dyslipidemia was defined as the presence of lipid-lowering drugs and/or low HDL (< 1.0 mmol/l) or high triglyceride (> 1.7 mmol/l) levels.
(iii) **Biochemical analysis.** Fasting total and HDL cholesterol, triglyceride, transaminase, high-sensitivity C-reactive protein (hs-CRP), leptin, FGF21, the steroid panel, estradiol (E2), SHBG, LH, and FSH levels were measured. Free testosterone (FT) was both directly measured by equilibrium dialysis and calculated via the Vermeulen Eq. (20).
(iv) **Transcriptomic analysis**. Total RNA extraction was performed with the PAXgene Blood RNA Kit (Qiagen, Hilden, Germany) following the manufacturer’s instructions. Two hundred nanograms of RNA per sample were sequenced on the DNBseq platform (BGI Europe A/S, Denmark), and the resulting FASTQ files were processed with the STAR alignment tool [21]. Analysis of the gene expression data was performed via iDEP (http://bioinformatics.sdstate.edu/idep) [22]. Hierarchical clustering of the 1000 most variable genes was based on correlation (complete linkage), followed by principal component analysis (PCA) of all ~ 14,000 detected genes. Genes driving specific principal components have been depicted based on correlation after Bonferroni multiple correction unless differently indicated. Differentially expressed genes (DEGs) were identified with DESeq2 (FDR 5%, fold change 1.5), and functional enrichment analysis of DEGs was performed with g:GOSt (https://biit.cs.ut.ee/gprofiler) [23] via the g:SCS (set count and sizes) algorithm for multiple correction. Blood transcriptomic data were available for 29 subjects (20 obese and nine lean). Furthermore, two ObHH and one ObnHH individuals were excluded because of extreme differential expression in pathways linked to the response to virus and immune processes, which is consistent with ongoing viral infections.

#### Protocol 2

Ten ObHH, eleven ObnHH and nine lean controls agreed to undergo additional phenotyping:

(i) **Targeted metabolomics**: nonesterified fatty acids (NEFAs) and sphingolipids, including ceramides, amino acids, and ketone bodies.
(ii) **Afternoon blood sample at 3 PM** for diurnal rhythm assessment of cortisol, FGF21, and NEFA.
(iii) **Brain MRI without contrast,** including diffusion tensor imaging (DTI) [24], is used to visualize the hypothalamic microstructure. Three ObHH patients encountered claustrophobic issues and could not complete the exam. One subject was excluded because of the incidental discovery of normal-pressure hydrocephalus. Finally, a fifth subject could not be explored because of a technical issue. In the end, MR images from five ObHH, eleven ObnHH, and nine lean subjects were analyzed.

During preparation for RYGB, 12 participants opted to defer it or to receive a different type of bariatric surgery. These subjects were included only for the baseline analysis. The remaining 20 subjects underwent a complete follow-up evaluation at 12 months (M12) after RYGB. VAT tissue biopsies were collected during surgery (see the Supplementary Appendix). Nine patients consented to a longitudinal follow-up with fasting blood samples at D2, D28, M3, and M6 post-RYGB.

### Biochemical measurements

Technical details regarding the serum/plasma measurements are available in the Supplementary Appendix. The total FGF21 protein concentration was measured via ELISA (Quantikine Kit—R&D Systems). The results of this kit have been shown to closely correlate with those of other kits that measure intact FGF21 [25]. Serum T and other steroid hormones were quantified via the ultrahigh-performance liquid chromatography (UHPLC)-mass spectrometry (MS) method (UHPLC-MS) [26]. Equilibrium dialysis followed by direct measurement of the FT in the dialysate was performed as previously reported [27].

### Statistical methods

Statistical analyses were performed via R 4.2.2 (R Foundation for Statistical Computing, Vienna, Austria) and GraphPad Prism 9.0 (GraphPad Software, Inc., San Diego, CA). For categorical variables, Fisher’s exact tests were performed. For continuous variables at baseline (three groups), one-way Kruskal‒Willis tests were conducted. A nonparametric test was favored because of the small sample size in the control group. The associations between reproductive and metabolic outcomes were evaluated via simple linear regressions. Pearson correlation coefficients (r) were calculated. To identify predictors of T levels in men with obesity, BMI, HOMA-IR, VAT, and hs-CRP were examined via multivariate linear regressions. The differences in variables before and 12 months after bariatric surgery were analyzed via paired Student’s t tests (or Wilcoxon signed-rank tests). For the longitudinal analysis in Protocol 2 (multiple timepoints after bariatric surgery), a linear mixed regression model was used. For each studied outcome, the model integrates two explicative variables: the time since bariatric surgery (categorical variable according to the timepoint, fixed effect) and the subject identity (random effect). A p value less than 0.05 was considered statistically significant. Bioinformatics analyses, including PCA, were performed via the open-source programming language and are detailed in the Supplementary Material. Unless otherwise indicated, results of correlations with omics data were adjusted using Benjamini–Hochberg false discovery rate (FDR) method.

## Results

### Testosterone levels are a key contributor to phenotypic variability in men with obesity

**Fig. 1:**
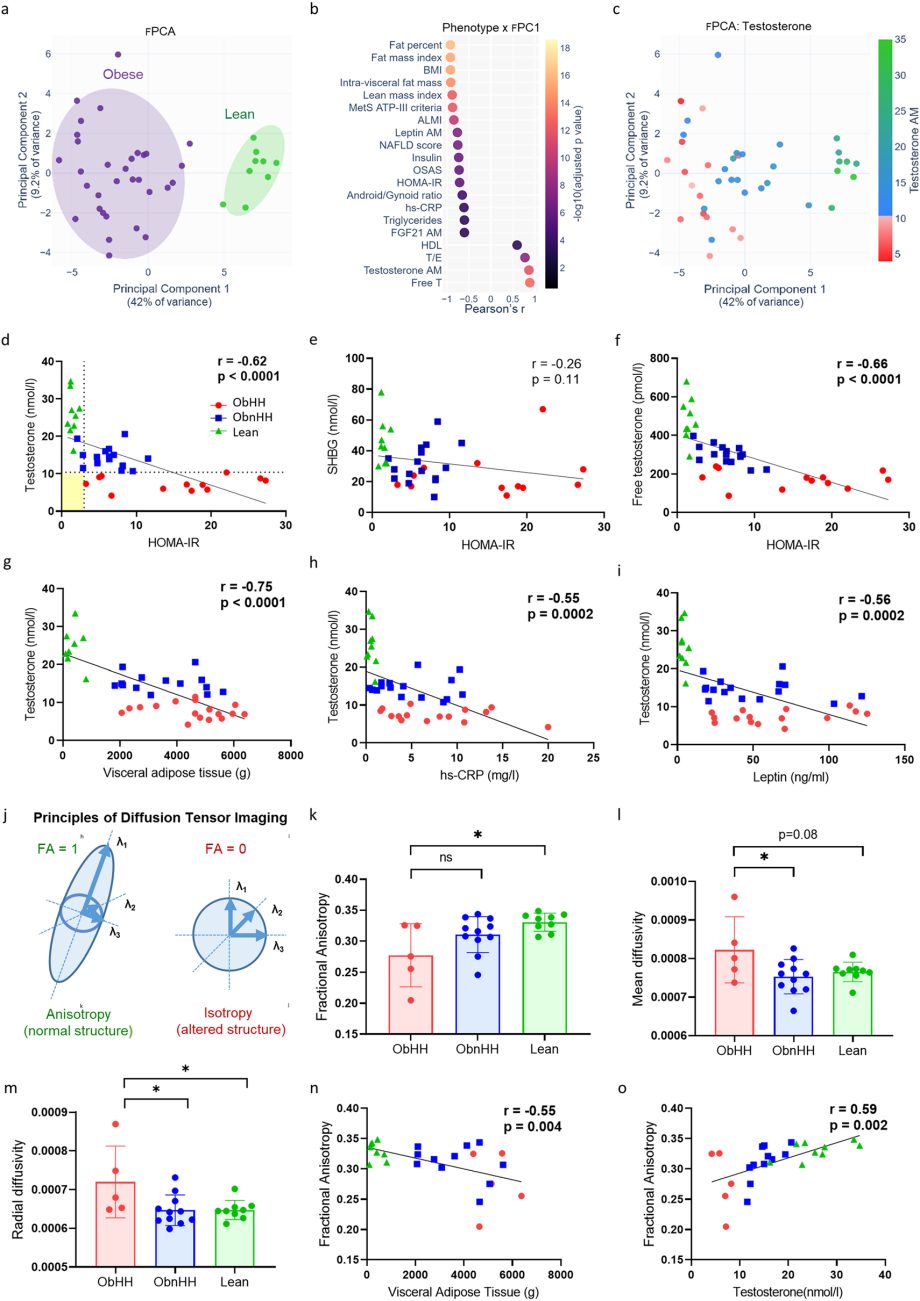
Hypogonadism in men with obesity is associated with worsening metabolic defects and hypothalamic alterations. **a** Scatter plot representing the first two principal components resulting from PCA of the metabolic and reproductive phenotypes of thirty-two obese patients and nine lean subjects. **b** Dot plot illustrating the contribution of individual variables (to patient phenotypic variance) measured as their correlation with principal component 1 (PC.1). **c** Overlay of PCA two first principal components with T levels (colorscale) illustrating a gradient of T along PC.1. **d** Scatter plot of the HOMA-IR index with T levels. The participants are represented as follows: Obese with HH (ObHH, red circles, n = 12), Obese without HH (ObnHH, blue squares, n = 16), and Lean (green triangles, n = 9). Missing values of HOMA-IR are due to hemolyzed blood samples in two patients (one ObHH and one ObnHH) and exogenous insulin therapy in two ObHH men. Regression lines, Pearson correlation coefficients (r) and related p values are shown. The light yellow rectangle reflects the inability to detect any patient with HH (T < 10.4 nmol/l, horizontal dashed line) and normal insulin sensitivity (HOMA-IR < 3.0, vertical dashed line). **e**, **f** Scatter plots of the HOMA-IR index with the levels of SHBG and FT as calculated by the Vermeulen formula. **g**–**i** Linear regression model showing the relationships between T levels and visceral adipose tissue (n = 39), serum hs-CRP (n = 40), and plasma leptin (n = 40) levels. **j** Principles of DTI illustrating that fractional anisotropy is a marker of preserved (high value, green) or altered structure (low value, red). k-m) Between-group comparisons of fractional anisotropy, mean diffusivity, and radial diffusivity levels (subjects included in Protocol 2; see Methods: Obese n = 16 vs Lean n = 9). **n**, **o** Linear regression model representing the relationship between fractional anisotropy vs visceral adipose tissue and serum T levels. * p < 0.05; ** p < 0.01; *** p < 0.001; **** p < 0.0001; *ns* not significant

PCA was applied to the phenotypic data of the whole cohort (fPCA, Table 1). Obese and lean subjects were clearly separated along the first principal component (fPC.1) (Fig. 1a), driven by adiposity indices and insulin resistance (HOMA-IR) (Fig. 1b). Notably, T levels clearly gradient across fPC.1, which is consistent with an inverse association with the severity of metabolic defects (Fig. 1b, c). Thus, obese subjects were further classified according to T levels using a cutoff of 10.4 nmol/l (300 ng/dl) [28]. When the previously published calculated FT cutoff of 220 pmol/l was used to define HH [29], the classification of the cohort remained largely unchanged (Figure S2b). The calculated FT values were highly correlated with the direct measurement of the FT by equilibrium dialysis (r = 0.95, p < 0.0001; Fig. S2a), confirming the use of the former as a valid surrogate.

**Table 1:** Baseline characteristics of the study participants according to body mass index and gonadal status

	Obese (n = 32)			Lean (n = 9)	P value Ob vs Lean	P value HH vs Non-HH
	All	HH (n = 15)	Non-HH (n = 17)			
Age (years)	45.1 ± 8.1	47.0 ± 8.5	43.4 ± 7.6	38.1 ± 12.5	0.14	0.24
*Anthropometrics*						
Body mass index (kg/m^2^)	44.7 ± 7.4	46.0 ± 7.7	43.5 ± 7.2	22.5 ± 2.2	< 0.0001	0.45
*Metabolic comorbidities*						
Hypertension	19/32	13/15	6/17	1/9	0.02	0.007
OGTT status						
Glucose intolerance	11/32	3/15	8/17	0/9	0.08	0.15
Type 2 diabetes	12/32	9/15	3/17	0/9	0.04	0.03
Dyslipidemia	19/32	12/15	7/17	2/9	0.07	0.04
Metabolic syndrome	22/32	13/15	9/17	0/9	0.0003	0.05
*Glucose metabolism*						
Glucose (mmol/l)	6.2 ± 1.1	6.7 ± 1.3	5.7 ± 0.6	5.3 ± 0.3	0.005	0.01
Insulin (mU/l)	37 ± 23	53 ± 27	26 ± 10	6 ± 2	< 0.0001	0.02
HOMA-IR	10.2 ± 7.2	15.2 ± 8.4	6.5 ± 2.6	1.5 ± 0.5	< 0.0001	0.01
Hb1Ac %	6.1 ± 1.0	6.4 ± 1.1	5.8 ± 0.8	–	–	0.01
*Adiposity-Adipokines*						
Leptin (ng/ml)	57 ± 34	65 ± 35	49 ± 32	4 ± 2	< 0.0001	0.15
Fat percentage (%)	46.2 ± 5.6	46.9 ± 5.9	45.5 ± 5.4	21.2 ± 5.2	< 0.0001	0.50
Visceral adipose tissue (g)	4083 ± 1387	4521 ± 1380	3671 ± 1302	368 ± 273	< 0.0001	0.09
*Lipids*						
Total cholesterol (mmol/l)	4.5 ± 0.8	4.3 ± 0.7	4.6 ± 0.8	4.1 ± 0.7	0.22	0.31
Triglycerides (mmol/l)	1.9 ± 0.9	2.3 ± 1.0	1.5 ± 0.7	0.8 ± 0.4	0.0003	0.01
HDL cholesterol (mmol/l)	1.1 ± 0.2	0.9 ± 0.1	1.2 ± 0.3	1.4 ± 0.4	0.02	0.002
*Liver steatosis*						
ALAT (IU/l)	44 ± 20	41 ± 16	47 ± 23	26 ± 13	0.0007	0.48
ASAT (IU/l)	32 ± 10	28 ± 8	35 ± 11	29 ± 8	0.53	0.05
NAFLD Fibrosis Score	−0.23 ± 1.19	−0.12 ± 1.01	−0.33 ± 1.35	−2.53 ± 0.67	< 0.0001	0.85
*Stress and inflammation*						
hsCRP (mg/l)	6.3 ± 4.6	7.7 ± 5.3	4.9 ± 3.6	0.6 ± 0.3	< 0.0001	0.10
Cortisol (nmol/l)	269 ± 95	276 ± 117	264 ± 73	381 ± 61	0.002	0.94
*Reproductive status*						
ADAM score, positive	14/21	7/9	7/12	1/9	0.01	0.36
Total testosterone (nmol/l)	11.2 ± 4.2	7.4 ± 1.7	14.5 ± 2.8	25.8 ± 5.8	< 0.0001	< 0.0001
SHBG (nmol/l)	28 ± 14	176 ± 42	299 ± 48	46 ± 15	0.002	< 0.0001
Calculated FT (pmol/l)	241 ± 76	23 ± 14	32 ± 13	484 ± 114	< 0.0001	0.009
LH (U/l)	5.2 ± 2.3	5.3 ± 2.4	5.2 ± 2.3	6.0 ± 3.3	0.80	0.78
FSH (U/l)	4.5 ± 3.2	5.1 ± 3.4	4.1 ± 3.1	4.7 ± 1.8	0.32	0.42
Estradiol (nmol/l)	0.14 ± 0.05	0.13 ± 0.05	0.14 ± 0.05	0.13 ± 0.05	0.85	1.0
Testosterone/Estradiol ratio	92 ± 44	63 ± 24	117 ± 42	209 ± 71	< 0.0001	0.0001

*OGTT* oral glucose tolerance test, *NAFLD* nonalcoholic fatty liver disease. T, testosterone. Metabolic syndrome was defined on the basis of the ATP-III criteria. Dyslipidemia was defined as the presence of lipid-lowering drugs and/or low HDL (< 1.0 mmol/l) or high triglyceride (> 1.7 mmol/l) levels. Free testosterone and NAFLD fibrosis scores were calculated on the basis of published algorithms [20, 55]. The data are shown as the means ± standard deviations. Between-group differences were analyzed via the Wilcoxon rank sum test for continuous variables and Fisher’s exact test for categorical variables

Compared with ObnHH, ObHH presented a greater incidence of hypertension and T2D, in addition to lower HDL and higher triglyceride levels (Table 1). These differences persisted after adjustment for age and BMI (Table S1). All HH subjects had altered insulin sensitivity with a relatively large spectrum, whereas eugonadal men with obesity presented normal or only modestly elevated HOMA-IR (Fig. 1d). The OGTT curves revealed more severe hyperglycemia and a distinct insulin pattern in ObHH, consisting of blunted early secretion followed by prolonged hyperinsulinemia (Fig. S3). Notably, SHBG levels exhibited weaker associations with HOMA-IR (Fig. 1e) and other metabolic markers both in the whole cohort and within the obese group (Fig. S4). Conversely, the associations of FT levels with metabolic outcomes were identical to those of total testosterone (Fig. 1f, Fig. S4). Furthermore, ObHH tended to have more VAT (Fig. 1g, Fig. S5) and higher serum levels of hs-CRP (Fig. 1h, Fig. S5) despite similar total FM and serum leptin levels compared with those of ObnHH (Fig. 1i, Fig. S5). HOMA-IR was the only significant predictor of low T after multivariate adjustment (Table S2).

### Brain MRI reveals altered hypothalamic structure in ObHH

Central inflammation was assessed via DTI. The principle of fractional anisotropy (FA) in DTI is shown in Fig. 1j. FA was significantly lower in ObHH than in lean (p = 0.01), whereas ObnHH did not differ from the controls (Fig. 1k). The mean and radial diffusivity were greater in ObHH than in ObnHH (Fig. 1l, m). Linear regression revealed a significant association of FA with VAT (Fig. 1n), serum T (Fig. 1o) and insulin resistance (Fig. S6).

### Blood transcriptomics reveals novel markers of metabolic defects

**Fig. 2:**
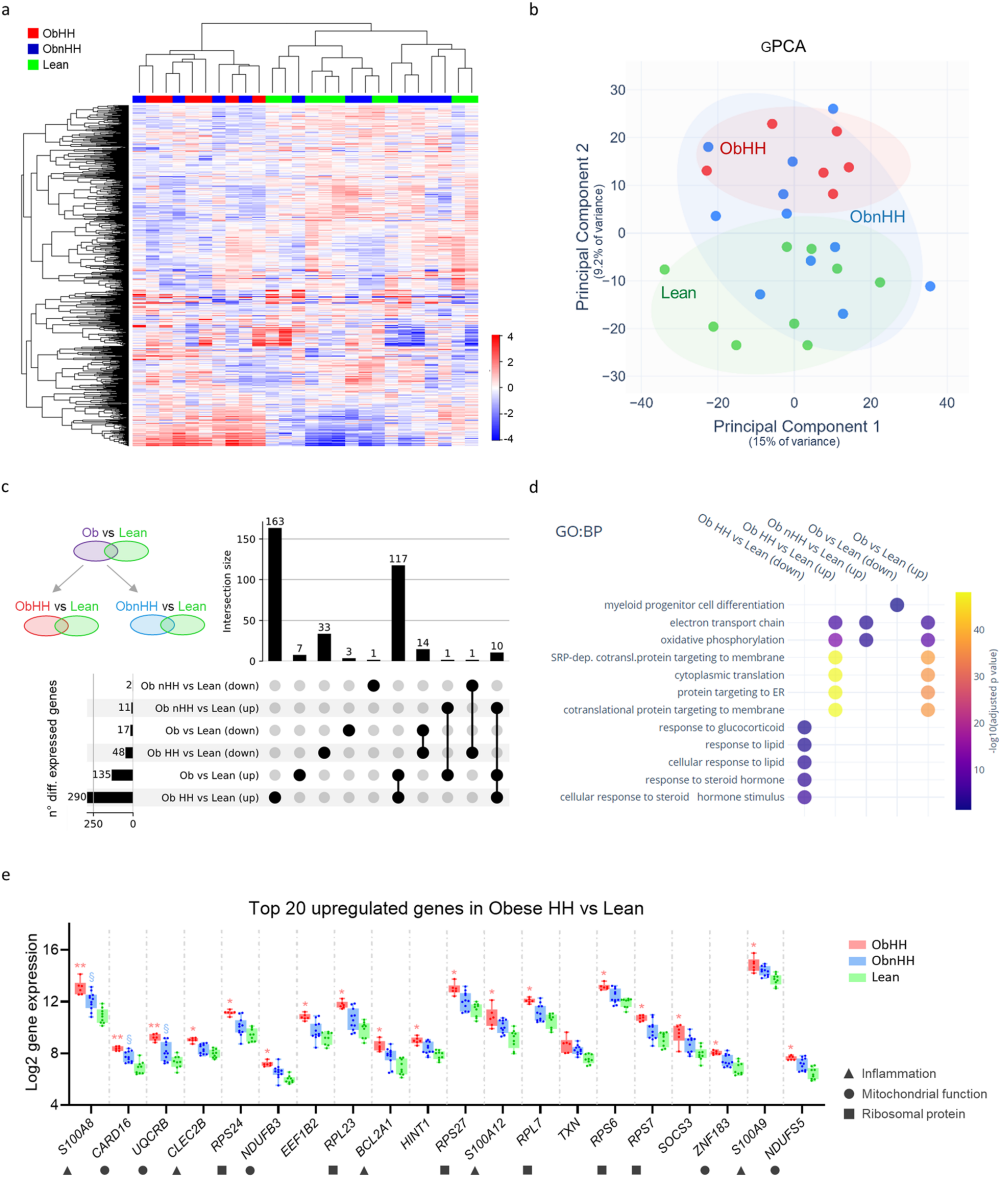
Hypogonadal and eugonadal men with obesity display different degrees of altered gene expression. **a** Heatmap representing the relative expression (Z score) of the top 1000 variable genes followed by hierarchical clustering on the basis of correlation (complete linkage). The centers of genes and samples were obtained by subtracting their means. **b** Scatter plot representing the first two principal components resulting from PCA on all detected genes. Obese patients with hypogonadotropic hypogonadism (ObHH, n = 6), obese patients without hypogonadotropic hypogonadism (ObnHH, n = 11) and lean controls (Lean, n = 9) are displayed in red, blue, and green, respectively. **c** UP plot illustrating the number of differentially expressed genes and their intersections according to the comparisons used in the differential gene expression analysis (upper left schematics). **d** Dot plot illustrating the enrichment of gene ontology biological processes (GO:BP) in differentially expressed genes. **e** Expression of the top 20 upregulated genes in obese HH patients versus lean controls. *p < 0.001, **p < 1.0 × 10^7^, ^§^p < 0.05

Hierarchical clustering on the basis of the expression of the most variable genes perfectly separated ObHH and Lean (Fig. 2a). Similar results were observed with PCA of the gene expression (gPCA) of all 14,000 detected genes. ObHH and lean were completely separated along the second principal component (gPC.2), whereas ObnHH failed to form an independent cluster (Fig. 2b). Head-to-head group comparisons were performed (Fig. 2c). Compared with lean, ObHH presented the greatest number of significant DEGs, most of which were exclusive to this pair (Fig. 2c). Functional enrichment analysis revealed that oxidative phosphorylation (OXPHOS, upregulated genes) and response to lipids (downregulated genes) were among the top affected biological processes (Fig. 2d). Finally, the majority of upregulated genes in ObHH vs Lean were linked to inflammation, mitochondrial homeostasis, or peptide biogenesis (i.e., ribosomal proteins) (Fig. 2e).

**Fig. 3:**
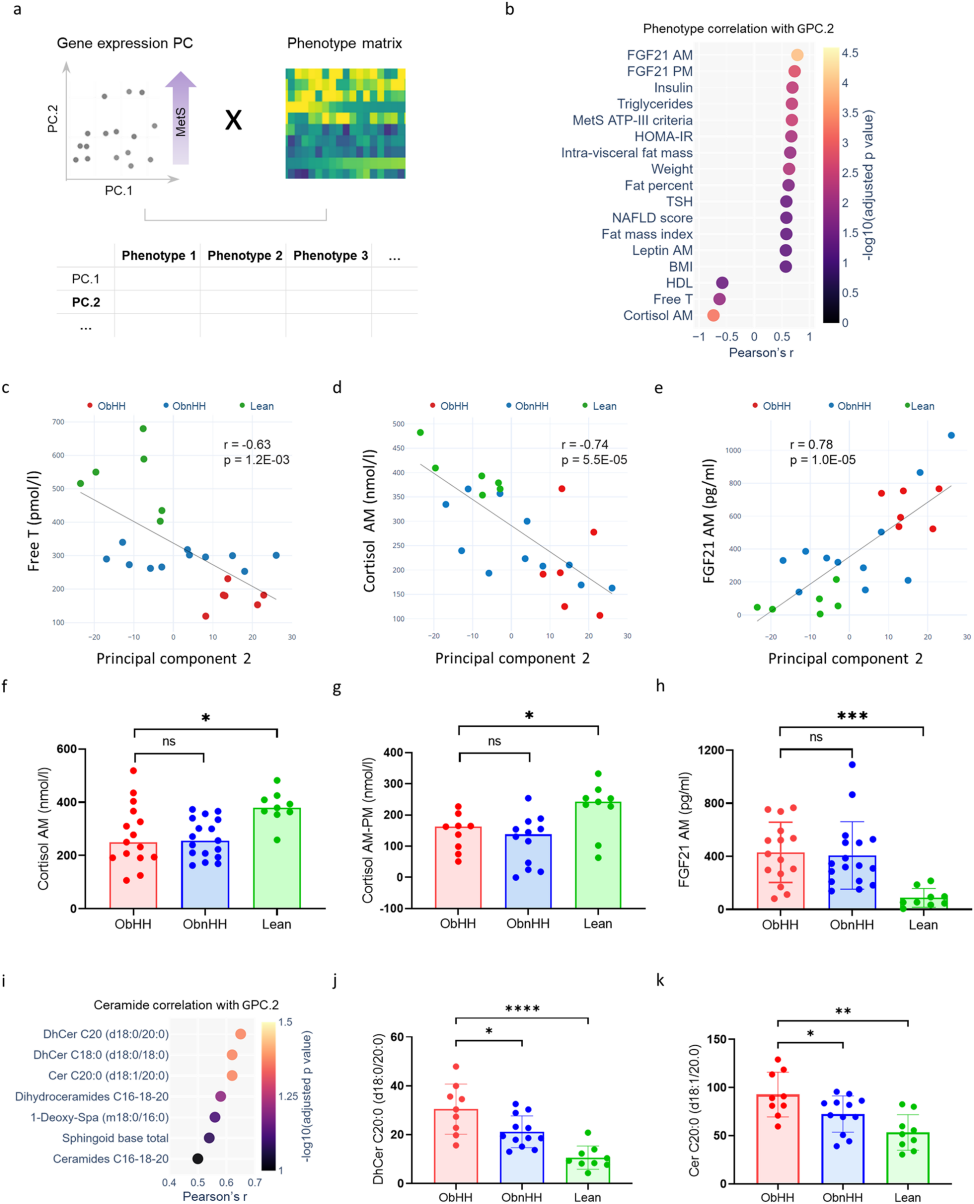
Transcriptomics highlights FGF21 and cortisol levels as markers of metabolic dysfunction beyond hypogonadism. **a** Schematics illustrating the strategy used to correlate individual phenotype variables with patient transcriptomic data in the form of principal component 2 from gene expression data (gPC.2). **b** Dot plot illustrating the correlation of individual phenotypic variables with patients’ transcriptomic data, measured as their correlation with gPC.2. **c**–**e** Scatter plots representing individual phenotypic variables such as FT (**c**), morning cortisol (**d**), and fasting FGF21 **e** as functions of gene expression PC.2. Obese patients with hypogonadotropic hypogonadism (ObHH), obese patients without hypogonadotropic hypogonadism (ObnHH), and lean controls (Lean) are displayed in red, blue, and green, respectively. Regression lines, Pearson correlation coefficients (r), and related p values are shown in each plot. **f**–**h** Between-group comparisons of morning (AM) serum cortisol levels (panel f; Protocol 1), diurnal variation in serum cortisol (by subtracting afternoon [PM] from morning values) levels (panel g; Protocol 2: Obese n = 21, Lean n = 9), and fasting plasma AM FGF21 levels (panel h, Protocol 1). **i** Dot plot illustrating the associations of individual ceramides with transcriptomic variability, evaluated as the correlations of individual plasma ceramide species with gPC.2. The top three classes were long acyl chain (C18 and C20) ceramides and dihydroceramides. **j**, **k** DhCer and Cer C20:0 levels, both of which were higher in ObHH than in both ObnHH and Lean; ****p < 0.0001; ***p < 0.001; **p < 0.01; *p < 0.05; *ns* not significant

To identify novel markers of metabolic defects, a correlation of gPC.2 with phenotypic variables was conducted (Fig. 3a). gPC.2 was strongly and positively associated with established markers of metabolic defects, including VAT, HOMA-IR, triglycerides and a number of MetS components, while it was inversely related to FT levels (Fig. 3b, c). Interestingly, the strongest correlations of gPC.2 were found with morning cortisol (r = −0.74, Fig. 3d) and fasting FGF21 (r = 0.78, Fig. 3e). Accordingly, both morning cortisol (8 AM) and delta cortisol (8 AM–3 PM values) were significantly lower in the obese groups than in the lean group (Fig. 3f-g), which is consistent with a disrupted diurnal rhythm. In contrast, plasma FGF21 levels were higher in obese individuals than in lean individuals (Fig. 3h). Further evidence supporting the link between MetS and gene expression status was the correlation between gPC.2 and the levels of saturated long acyl chain ceramides and dihydroceramides (C18 and C20) (Fig. 3i), which are known to induce insulin resistance and exert the worst metabolic effects among sphingolipids.[30] These specific species were significantly more common in ObHH than in both ObnHH and Lean and strongly correlate with key metabolic and reproductive markers (Fig. 3j–k, Fig. S7-S8).

**Fig. 4:**
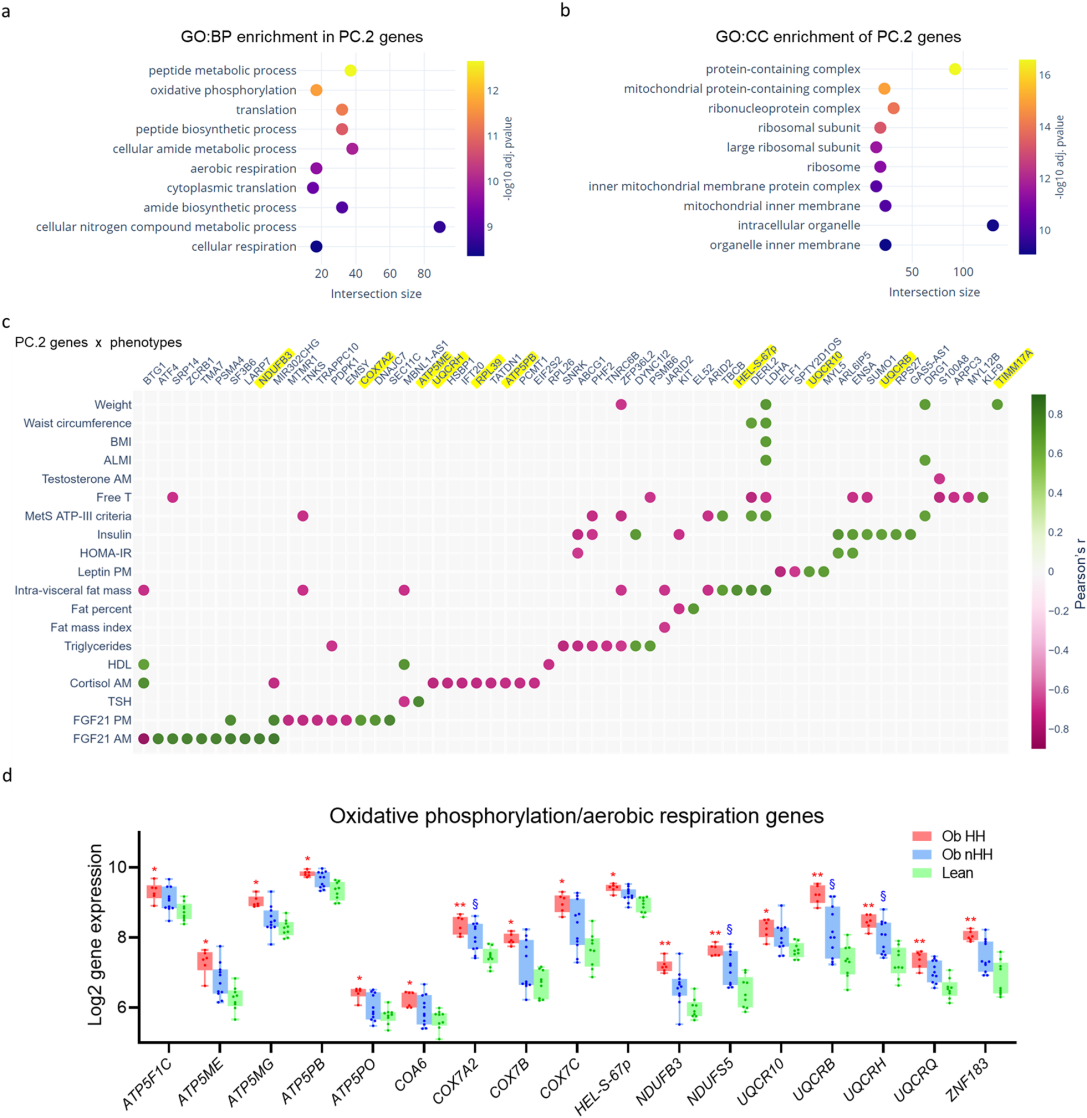
Genes involved in peptide metabolic processes, oxidative phosphorylation and aerobic respiration correlate with the phenotypic signatures of metabolic syndrome. Functional enrichment analysis of Principal Component 2 (PC.2) genes in the Gene Ontology database reveals enriched biological processes **a** and cellular components **b** linked with cellular metabolism. **c** Dot plot illustrating the correlation of individual phenotypic variables with the expression of PC.2 genes from patients’ transcriptomic data. The genes involved in oxidative phosphorylation/aerob*ic respiration are highlighted in yellow.* **d** Expression of PC.2 genes involved in oxidative phosphorylation/aerobic respiration in both the obese groups and lean controls. *p < 0.05 and **p < 1.0 × 10^–4^ (ObHH vs Lean); § p < 0.05 (ObnHH vs Lean)

Finally, genes driving gPC.2 were evaluated more closely. Functional enrichment analysis revealed a significant overrepresentation of biological processes, cellular components and signaling pathways critical for metabolic health (e.g., OXPHOS, translation, and mitochondria, insulin signaling) (Fig. 4a–d, Fig. S9). A gene‒phenotype correlation matrix revealed that the gPC.2 gene drivers correlated with at least one clinical variable (Fig. 4c, Table S3). FGF21 and cortisol were correlated with the greatest number of genes (Table S3), including two shared loci, *BTG1* and *MIR302CHG* (Fig. 4c).

### Reversal of metabolic defects following RYGB independent of HH status

**Fig. 5:**
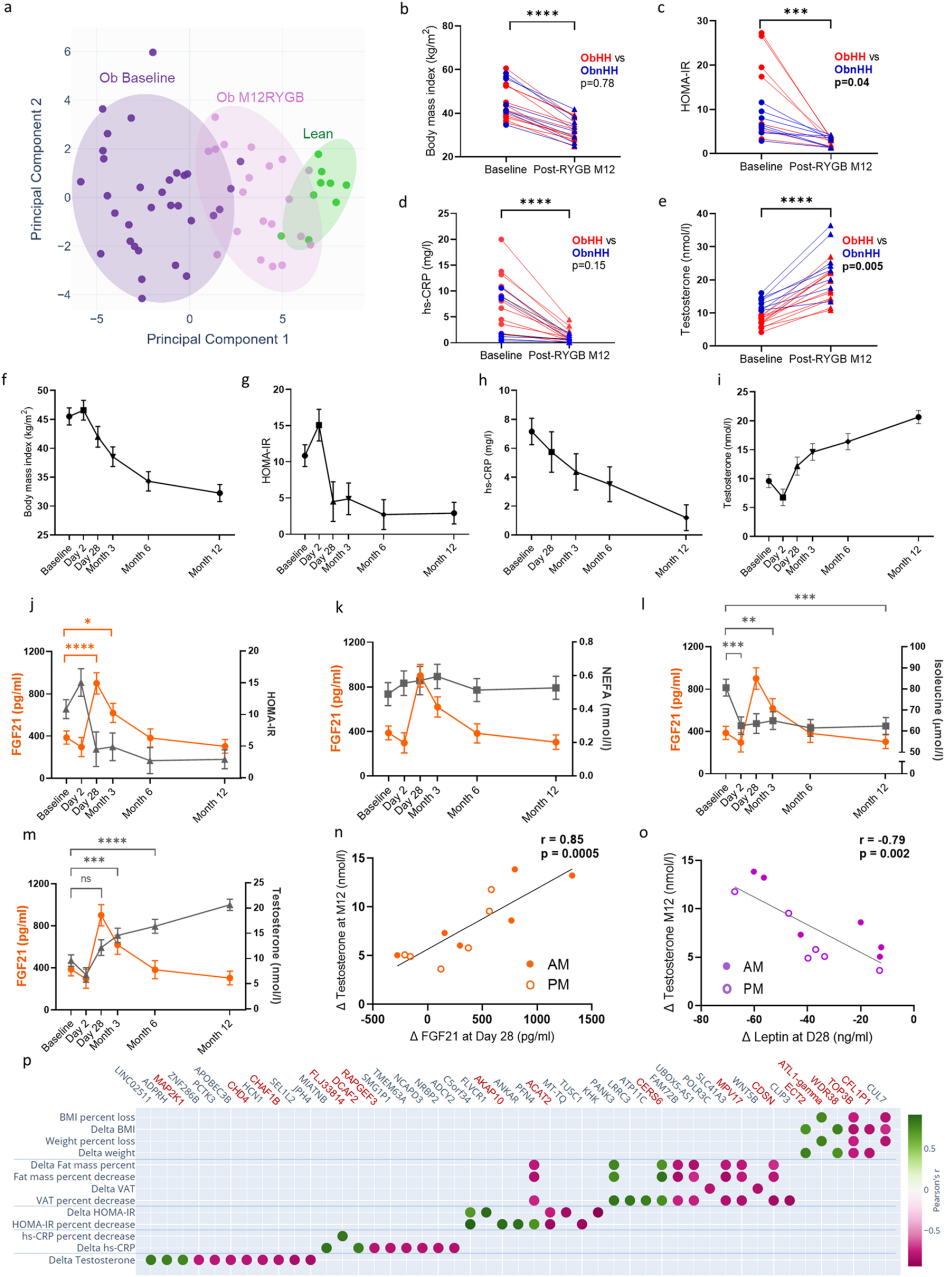
Metabolic and reproductive improvements following bariatric surgery are closely associated with early increases in plasma FGF21 levels. **a** RYGB induced significant phenotypic changes, as illustrated by a rightward shift along Principal Component 1 (PC.1) from phenotypic PCA (fPCA). Compared with the baseline obese group (dark purple, n = 32), men at month 12 (M12) post RYGB (light purple, n = 20) were healthier (green, n = 9). **b**–**e** Changes in BMI, HOMA-IR, hs-CRP, and serum T levels throughout one year post-RYGB in 20 men with obesity. Each line corresponds to an individual patient (obese with hypogonadotropic hypogonadism [ObHH] and obese without hypogonadotropic hypogonadism [ObnHH] in red and blue, respectively). Brackets show the extent of the difference between the baseline and M12 post-RYGB groups as a whole. In addition, the comparison of the relative percent change in these outcomes according to the gonadal status at baseline is shown in the upper right quadrant of each graph. **f**–**i** Longitudinal evolution of the same parameters in nine participants who consented to multiple post-RYGB visits via linear mixed model regression. Owing to missed visits, data were available for eight participants on Day 2 and Month 3 and for six participants on Day 28. All four parameters showed significant changes beginning on Day 28. **j** Post-RYGB FGF21 changes (orange line, left Y axis) overlaid with HOMA-IR shifts (gray line, right Y axis). Brackets in orange illustrate the timepoints with significant differences in plasma FGF21 levels compared with those at baseline. **k**–**l** Temporal associations of post-RYGB FGF21 changes in orange (left Y-axis) with serum nonesterified fatty acid (NEFA, panel k) and plasma isoleucine levels (panel l), both of which are shown in gray (right Y-axis). m) Superimposition of FGF21 changes (orange line, left Y axis) and recovery of serum T levels (gray line, right Y axis). **n**, **o** Linear regression model representing the relationship of the postbariatric change in plasma FGF21 and leptin levels from baseline to Day 28 with the change in serum T levels from baseline to M12. For each outcome, the difference (Δ, delta) was calculated for both fasting at 8 AM (full circles) and afternoon samples at 3 PM (empty circles). **p** Dot plot illustrating the top-ranked correlation relationships of clinical variables reflecting post-RYGB recovery with VAT gene expression. The genes with known associations with metabolic or reproductive traits are shown in red. The results in graphs f-m) are shown as the means ± standard errors. ****p < 0.0001; ***p < 0.001; **p < 0.01; *p < 0.05; *ns* not significant

Men with obesity lost 20.9 to 39.1% of their initial weight at M12 post-RYGB (Table S4). The surgery resulted in a shift along the fPC.1 toward healthy subjects (Fig. 5a). Weight loss did not differ between ObHH and ObnHH (Fig. 5b). As expected, drastic improvements in insulin sensitivity (HOMA-IR, Fig. 5c) and low-grade inflammation (hs-CRP, Fig. 5d) were observed, which was consistent with the resolution of metabolic defects in the majority of patients (Table S4). In parallel, the serum T levels more than doubled to reach the normal range (> 10.4 nmol/l) in all the subjects at M12 (Fig. 5e). Notably, ObnHH also presented a mean 90% increase in T levels. Hypothalamic structure was reassessed via MRI-DTI at M12 post-RYGB. FA increased in five out of the six patients with available MRI data post-RYGB, but the difference was not statistically significant (Fig. S10). In this small sample, the changes in the mean and radial diffusivity were less consistent.

### The unique trajectory of FGF21 after RYGB is tightly linked with metabolic improvements

To characterize the pace and sequence of post bariatric changes, a subset of patients had multiple visits. Weight loss was rapid until M6, at a slower rate thereafter (Fig. 5f). The drop in HOMA-IR and leptin levels was sharp during the first month, when only moderate weight loss had occurred (Fig. 5g**,** Fig. S11), whereas the reduction in hs-CRP followed a more gradual pattern (Fig. 5h). Fasting plasma FGF21 tended to decrease by M12 (372 ± 186 vs 297 ± 195 vs pg/ml, p = 0.07). In the longitudinal study with multiple visits, a peak in FGF21 (2.5-fold increase, p = 0.04) at D28 post-RYGB concomitant with a decrease in HOMA-IR (Fig. 5j) was detected. FGF21 was not elevated at D2 post-RYGB, suggesting a link with acute surgery-related stress. Among the known stimuli of FGF21 release, a gradual increase in fasting NEFA levels occurred, although this increase was not significant (Fig. 5k), whereas serum β-hydroxybutyrate levels varied widely (Fig. S11). Notably, branched-chain amino acid (BCAA) and, in particular, isoleucine levels decreased sharply at D2 (p = 0.01) prior to the FGF21 peak (Fig. 5l, Fig. S12).

### FGF21 peak levels predict HH reversal post-RYGB

The predictors of T levels post-RYGB were then evaluated. The T levels started to recover by D28, with a steady increase thereafter (Fig. 5i). Notably, the FGF21 peak preceded HH reversal (Fig. 5m). Delta FGF21 and leptin levels from baseline to D28 were strongly associated with delta T from baseline to M12 (r = 0.85, p = 0.0005 and r = −0.79, p = 0.002, respectively) (Fig. 5n, o), but this was not the case for other metabolic parameters (Fig. S13).

### VAT transcriptomics predicts weight loss post-RYGB

VAT transcriptomes were available for 16 patients (nine with ObHH and seven with ObnHH). We found a significant correlation between VAT gene expression and changes in key clinical variables (BMI, weight, FM, VAT, HOMA-IR, CRP, and T) from baseline to M12 post-RYGB (Fig. 4p, Table S5). These genes are involved in biological processes linked with energy homeostasis, including ceramide production (*CERS6* [31]) and thermogenesis (CDNS [32]). Within the top 50 associations, we found several genes previously identified in genome-wide association studies (GWASs) for obesity and other metabolic traits (Table S5).

## Discussion

Our study uses a multiomics approach to elucidate the heterogeneity of obesity in men. Testosterone was confirmed to be a key contributor to phenotypic variability. After multivariate adjustment, only the association of T with HOMA-IR persisted, suggesting that insulin resistance is the predominant player in metabolic‒reproductive cross talk. Consistently, low T has been previously associated with insulin resistance [33] and the occurrence of T2D [34]. Furthermore, Mendelian randomization revealed that higher T levels reduce T2D risk in men [35]. In contrast to previous epidemiologic data suggesting that SHBG is more strongly associated with metabolic risk in men than T or FT is [28], we found a much weaker link between SHBG and the metabolic phenotype of severe obesity in males (BMI > 35 kg/m^2^). This finding is in line with a previous meta-analysis showing that the relationship between SHBG and MS is mostly strong in overweight but not severely obese men [36]. Notably, a large spectrum of metabolic changes was observed within the ObHH, indicating that the metabolic variability in obesity is not entirely explained by serum T levels.

The link between MetS and hypogonadism was further confirmed by the marked increase in serum T levels following RYGB. HH men exhibited greater metabolic benefits post-RYGB, which is in agreement with the findings of a previous study reporting a greater decrease in waist circumference in ObHH men [37]. The upward shift in T levels also occurred in eugonadal obese males, questioning whether the currently proposed lower normal range for serum T is accurate in men without metabolic dysfunction. Previously, a threshold of 12.1 nmol/l (2.5 percentile) was established via LC‒MS in a study of healthy young nonobese (BMI < 30 kg/m^2^) males selected from a community-dwelling setting [38]. However, metabolic defects such as insulin resistance that could have lowered T levels were not systematically assessed in that study.

Our study revealed that hypothalamic MRI is an effective tool for assessing central inflammation in men with obesity, as previously suggested in retrospective observations [39]. In particular, ObHH patients exhibited lower fractional anisotropy and higher diffusivity—two DTI patterns consistent with hypothalamic inflammation—than both eugonadal obese and lean men did. Visceral adiposity was the only metabolic marker associated with both hypothalamic injury and T levels, in line with the previously reported role of saturated fatty acids as the main trigger of hypothalamic inflammation in rodents [40]. In the limited number of MRI pairs—pre-RYGB and post-RYGB—fractional anisotropy tended to increase. Larger studies are needed to determine whether hypothalamic inflammation is reversible after weight loss.

Obesity is known to modify gene expression in a variety of tissues. In agreement with previous observations [41, 42], our results confirmed the upregulation of critical genes involved in inflammation, OXPHOS, and protein synthesis. In addition, we demonstrated that, compared with obesity alone, hypogonadism has a more profound transcriptomic impact. Conversely, obese men without hypogonadism exhibit more heterogeneous gene expression, spanning between the transcriptomic profile of ObHH and lean controls. The upregulation of OXPHOS genes in blood apparently contrasts with a prior study of muscle transcriptomics showing downregulated OXPHOS genes in middle-aged obese men [33]. However, other studies also reported the upregulation of OXPHOS genes in the livers of diabetic obese individuals [43], as well as in the peripheral blood of abdominally obese individuals [44]. This discrepancy could be due in part to tissue-specific adaptations to metabolic alterations.

Through the combination of gene expression and phenotyping data, we highlighted gPC2 as the transcriptomic signature of high metabolic risk. Furthermore, cortisol and FGF21 emerged as the top clinical parameters associated with gPC2, supporting their potential role in metabolic risk stratification. Accordingly, men with obesity had lower serum cortisol and higher plasma FGF21 levels than lean controls did, independent of their HH status. Low morning cortisol challenges the old dogma that obesity is a state of chronic cortisol excess [45]. Notably, our observation of a blunted cortisol diurnal rhythm is in agreement with a recent study showing impaired cortisol cyclicity in men with a BMI > 40 kg/m^2^ [46]. The paradoxical finding of high serum FGF21 levels in men with obesity is consistent with a state of FGF21 resistance, as shown in mice [6, 47], similar to what occurs with leptin.

FGF21 and cortisol are both associated (in opposing directions) with the expression of 2 genes: (i) *BTG1,* a member of the B-cell translocation antiproliferative family, which is implicated (when overexpressed) in liver steatosis regression [48], and (ii) *MIR302HCG*, a long noncoding host gene that regulates the transcription of the MIR302 family, which in turn enhances central insulin signaling in neurodegenerative disorders [49]. *S100A8*, a member of the S100 calgranulin family, is the only gene highly correlated with both T and FT, and its protein is known to be an active promoter of inflammation [50]. These data support the role of low-grade systemic inflammation in the pathogenesis of obesity-induced HH. Finally, several gene expression markers in VAT, a key player in MetS, could efficiently predict the degree of phenotypic recovery post-RYGB. The strength of these data was attested by overlap with several genes previously linked to obesity in GWASs (see Table S5).

In our longitudinal study, we identified a sharp increase in FGF21 early after surgery concomitant with a decrease in insulin resistance and serum leptin levels. This FGF21 surge is not observed after diet-induced weight loss [14, 51] and could thus explain the greater metabolic benefits of RYGB in terms of mitochondrial function [52] and thermogenesis [53]. Interestingly, we detected a very early decrease in isoleucine levels post-RYGB. Indeed, marked protein restriction, particularly a reduction in BCAA levels, strongly activates FGF21 expression in mice [54] and could contribute to the FGF21 surge observed in our study. Finally, the increase in FGF21 by month 1 was a reliable predictor of post-RYGB serum T recovery. This finding is in line with our previous finding that Fgf21 stimulates GnRH secretion in median eminence explants [10].

This work has several limitations. Owing to the observational longitudinal design of the study, we cannot infer causality on the basis of these results. Indeed, some of the studied metabolic parameters (for instance, insulin resistance and low-grade inflammation) are interlinked, rendering difficult to determine whether observed changes are directly linked to RYGB or through indirect effects. Furthermore, although a strength of this study is the deep phenotyping, we included a relatively small sample size, in particular as regards to lean controls as well as the longitudinal post-RYGB follow-up. Lastly, physical activity status was not taken into account as a potential modifier of post-bariatric changes.

## Conclusions

In summary, this work highlights the key role of testosterone as a predictive parameter for metabolic health in men and challenges the accuracy of the current biochemical definition of hypogonadism in men with obesity. Testosterone deficiency correlates with visceral adiposity, insulin resistance, systemic and hypothalamic inflammation, as well as pronounced transcriptomic and metabolomic changes. Furthermore, integrated multi-OMICs analysis identified high FGF21 and low cortisol levels as novel predictors of metabolic dysfunction in obese individuals beyond the effect of hypogonadism.

## Supplementary Information

Supplementary Material 1

## Data Availability

Individual participant data reported in this article after deidentification (text, tables, figures, and appendices) will be shared with researchers who provide a methodologically sound proposal to achieve the aims of the suggested proposal. Proposals should be directed to nelly.pitteloud@chuv.ch. To gain access, data requestors need to sign a data access agreement.

## Abbreviations

BCAAs: Branched-chain amino acids
BMI: Body mass index
CER-VD: Ethics Committee for Research of the Canton of Vaud
D2: Day 2
D28: Day 28
DEGs: Differentially expressed genes
DTI: Diffuse tensor imaging
E2: Estradiol
FGF21: Fibroblast growth factor-21
FSH: Follicle-stimulating hormone
FT: Free Testosterone
GWAS: Genome-wide association studies
HH: Hypogonadotropic hypogonadism
HOMA-IR: Homeostatic model assessment for insulin resistance
HPG: Hypothalamic-pituitary–gonadal
Hs-CRP: High-sensitivity C-reactive protein
LH: Luteinizing hormone
M3: Month 3
M6: Month 6
M12: Month 12
MetS: Metabolic syndrome
NEFA: Nonesterified fatty acids
ObHH: Obese with Hypogonadotropic Hypogonadism
ObnHH: Obesity without hypogonadotropic hypogonadism
OGTT: Oral glucose tolerance test
PCA: Principal component analysis
RYGB: Roux-en-Y-gastric bypass
SHBG: Sex-hormone binding globulin
T: Testosterone
T2D: Type 2 diabetes
UHPLC‒MS: Ultrahigh-performance liquid chromatography‒mass spectrometry
VAT: Visceral adipose tissue
