# Supplementary material for: Multiomics unravels the complexity of male obesity: a prospective observational study
Source: J Transl Med. 2025 Jan 30;23:138. doi: 10.1186/s12967-024-06040-7 (PMC11783726; doi:10.1186/s12967-024-06040-7)
Supplement: Supplementary file 1 — Supplementary Material 1 [file 12967_2024_6040_MOESM1_ESM.docx]

**Multiomics unravels the complexity of male obesity: a prospective observational study**

**SUPPLEMENTARY MATERIAL**

Table of Contents

[**1.** **METHODS** 3](#_Toc183956386)

[**1.1** **Bariatric surgery** 3](#_Toc183956387)

[**1.2** **Visceral adipose tissue biopsies** 3](#_Toc183956388)

[**1.3** **Brain MRI – Diffuse Tensor Imaging** 3](#_Toc183956389)

[**1.4** **Biochemical measurements** 3](#_Toc183956390)

[**1.5** **Targeted metabolomics** 4](#_Toc183956391)

[**1.6** **Bioinformatics analysis** 5](#_Toc183956392)

[**1.7** **Sensitivity analysis** 5](#_Toc183956393)

[**2.** **SUPPLEMENTARY FIGURES** 6](#_Toc183956394)

[**2.1** **Figure S1. Flow chart of the study** 6](#_Toc183956395)

[**2.2** **Figure S2. Free testosterone by calculation versus direct measurement and impact on the classification of the obese cohort** 7](#_Toc183956396)

[**2.3** **Figure S3. Glucose and insulin changes following a standard 2h oral glucose tolerance test** 8](#_Toc183956397)

[**2.4** **Figure S4: Linear regression of metabolic parameters with testosterone, SHBG and free testosterone in the obese cohort** 9](#_Toc183956398)

[**2.5** **Figure S5. Metabolic phenotype according to obesity and reproductive status** 10](#_Toc183956399)

[**2.6** **Figure S6: Linear Regression of insulin resistance index and serum leptin levels with fractional anisotropy** 11](#_Toc183956400)

[**2.7** **Figure S7: Total and specific fatty acyl chain length ceramides and other sphingolipids** 12](#_Toc183956401)

[**2.8** **Figure S8: Linear regression of ceramides and selected dihydroceramides and deoxysphingolipids with insulin resistance index, free testosterone levels and FGF21** 13](#_Toc183956402)

[**2.9** **Figure S9: Functional enrichment analysis of GPC.2 correlating genes related signaling pathways** 14](#_Toc183956403)

[**2.10** **Figure S10: Change in fractional anisotropy in six patients with available diffusion-tensor Imaging MRI at baseline and month 12 post RYGB** 15](#_Toc183956404)

[**2.11** **Figure S11: Longitudinal changes post RYGB in key metabolic and reproductive outcomes** 16](#_Toc183956405)

[**2.12** **Figure S12: Longitudinal changes post RYGB in fasting plasma levels of branched-chain amino acids** 18](#_Toc183956406)

[**2.13** **Figure S13: Change in metabolic markers as potential predictors of the rise of serum T levels at month 12 post RYGB** 19](#_Toc183956407)

[**3.** **SUPPLEMENTARY TABLES** 20](#_Toc183956408)

[**3.1** **Table S1: Differences of obese groups in baseline characteristics after adjusting for age or body mass index** 20](#_Toc183956409)

[**3.2** **Table S2: Multivariate analysis of the effect of metabolic parameters on total and free testosterone of obese men** 22](#_Toc183956410)

[**3.3** **Table S3: Genes drivers of principal component 2 of blood transcriptomics** 23](#_Toc183956411)

[**3.4** **Table S4: Phenotypic changes in twenty morbidly obese men at month 12 after Roux-en-Y gastric bypass** 54](#_Toc183956412)

[**3.5** **Table S5: Top ranked correlation relationships of clinical variables describing post-RYGB recovery with VAT gene expression** 56](#_Toc183956413)

[**3.6** **Table S6: Comparison of characteristics of obese that consented vs declined protocol 2** 61](#_Toc183956414)

[**4.** **REFERENCES** 62](#_Toc183956415)

# **METHODS**

## **Bariatric surgery**

Eligible patients were initially scheduled to undergo RYGB. Following the inclusion of 32 obese patients and during the preparation for surgery, twelve participants opted to defer the RYGB or to receive a different type of bariatric surgery such as sleeve gastrectomy. These patients were included only for the baseline analysis. Eventually, twenty participants underwent laparoscopic RYGB with creation of a 15–20 ml gastric pouch, a 100–150 cm Roux limb, and a 30–50 cm biliopancreatic limb as previously described ^1^. Concomitant cholecystectomy was done in half of them due to detection of gallstones at pre-surgery ultrasound.

## **Visceral adipose tissue biopsies**

At the beginning of RYGB surgery, targeted biopsies of visceral fat were sampled. Fat specimens were immediately cleansed with NaCl. The tissue was subsequently dried with sterile compresses. The VAT samples were then wrapped in RNAase-free aluminum papers and quickly (less than 2 minutes) snap-frozen in liquid nitrogen to ensure immediate congelation. The fat specimens were later cut into smaller pieces on dry-ice in our laboratory before being stored in 2 ml tubes at -80°C.

## **Brain MRI – Diffuse Tensor Imaging**

A non-invasive imaging study (without contrast) with diffusion tensor imaging (DTI) ^2^. A T1 weighted image sequence was acquired then pre-processed using FreeSurfer software (<https://surfer.nmr.mgh.harvard.edu/fswiki>). After hypothalamus segmentation and diffusion weighted images processing, scalar maps were extracted from DTI as reviewed by Hagmann *et al* ^3^. Briefly, DTI is an MRI technique that measures macroscopic axonal organization in the brain by using the diffusion of weight molecules to generate contrast in magnetic resonance images. The two main derived parameters are fractional anisotropy (FA) and mean diffusivity. FA reflects the degree to which the diffusion of water molecules follows one versus many directions. For instance, white matter tracts normally exhibit a high degree of anisotropy due to their linear arrangement and the preferential diffusion of water along the long axis of the myelinated fibers ^2^.

## **Biochemical measurements**

Metabolic syndrome was defined based on the ATP-III criteria ^4^. Dyslipidemia was defined as the presence of lipid-lowering drugs and/or low HDL (< 1.0 mmol/l), or high triglycerides (> 1.7 mmol/l) levels. Free testosterone and NAFLD fibrosis score were calculated based on published algorithms ^5,6^. Glucose, high-sensitivity CRP, and other chemistry values were analyzed using commercially available assays (Cobas 8000, Roche Diagnostics). HOMA-IR was calculated according to the formula (glucose in mmol/l × insulin in mU/L)/22.5. Plasma LH, FSH, estradiol, TSH, and insulin were assessed by an electro-chemiluminescence immunometric assay (Cobas e 801, Roche Diagnostics). SHBG was measured using an enzyme immunoassay (Immulite 2000 XPi, Siemens) with a maximum intra-assay CV of 5.3%. Glycosylated hemoglobin (HbA1c) was measured using high-performance liquid chromatography (D-100, Bio-Rad Laboratories AG) and a maximum intra-assay and inter-assay CV of 1.1% and 1.6%, respectively. Serum total testosterone and other steroid hormones were quantified using a previously described and validated ultra-high-performance liquid chromatography (UHPLC) – mass spectrometry (MS) method ^7^. Ketone bodies and in particular b-hydroxybutyrate concentrations were determined on a Cobas Mira Plus (Roche Diagnostics, Switzerland) by an enzymatic photometric method adapted in house from the technique previously described by Ruell and Gass *et al* ^8^. For this measurement, 1 ml of venous blood was added in a previously cooled tube containing perchloric acid 0.6 N to deproteinize the sample. The interassay CV was 1.9% at 51.5 µmol/l and 1.7% at 148.9 µmol/l. Growth factors FGF21, FGF19, and Leptin were analyzed by ELISA (Quantikine ELISA kit - R&D Systems) with sensitivities of 1.61 pg/mL, 0.53 pg/mL, and 7.8 pg/mL, intra-assay CV of 2.9-3.9%, 3.6-6.4% and 3.0-3.3%, as well as inter-assay CV of 5.2-10.9%, 4.5-5.5%, and 3.5-5.4% respectively. Non-esterified fatty acids were measured with an enzymatic colorimetric method using the WAKO kit (WAKO Chemicals Inc. Richmond, VA, USA) with an intra-assay variation of 1%, inter-assay variation of 4–15%, and a detection limit of 0.01 mmol/L. Serum total adiponectin levels were assessed with the human Adipokine Magnetic Bead Panel 1 (Milliplex Map Kit, Cat # HADK1MAG-61K) with a sensitivity of 21 pg/ml and an intra-assay CV <15% and an inter-assay CV <10%. This kit is based on the Luminex technology ^9^.

## **Targeted metabolomics**

Targeted metabolomics for the quantification of a wide panel of sphingolipids (n=60 species) and amino acids (n=38 species) was performed by UHPLC-MS using validated methodology.

For sphingolipids, plasma samples (25 µL) were extracted by the addition of 100 µL of methanol spiked with the stable isotope-labelled (i.e., deuterated) internal standards (14 species overall). Following vortexing, the extracts were centrifuged for 15 minutes at 4000 g at 4°C and the resulting supernatants were collected for the liquid chromatography - tandem mass spectrometry (LC-MS/MS) analysis. Sphingolipids were quantified by LC-MS/MS analysis in positive ionization mode using a 6495 triple quadrupole system (QqQ) interfaced with 1290 UHPLC system (Agilent Technologies), adapted from Checa *et al* ^10,11^. Briefly, the chromatographic separation was conducted in a Zorbax Eclipse plus C8 column (1.8 μm, 100 mm × 2.1 mm I.D) (Agilent technologies). Mobile phase was composed of A = 5 mM ammonium formate and 0.2 % formic acid in water and B = 5 mM ammonium formate and 0.2% formic acid in MeOH at a flow rate of 400 μL/min. Column temperature was 40°C and sample injection volume 2 µL. The linear gradient elution starting from 80% to 100% of B (in 8 min) was applied and held until 14 min. The column was then equilibrated to initial conditions. ESI source conditions were set as follows: dry gas temperature 230 °C, nebulizer 35 psi and flow 14 L/min, sheath gas temperature 400°C and flow 12 L/min, nozzle voltage 500 V, and capillary voltage 4000 V. Dynamic multiple reaction monitoring was used as acquisition mode with a total cycle time of 500 ms. Optimized collision energies for each metabolite were applied. Raw LC-MS/MS data was processed using the Agilent quantitative analysis software (version B.07.00, MassHunter Agilent technologies). For absolute quantification, calibration curves and the stable isotope-labelled internal standards (IS) were used to determine the response factor. Linearity of the standard curves was evaluated for each metabolite using a 12-point range, in addition, peak area integration was manually curated and corrected when necessary.

For amino acid quantification, plasma samples (20 µL) were extracted by the addition of 30 µL of acidified water (0.1 % FA) and 250 µL of methanol spiked with the stable isotope-labelled internal standards. The extracted supernatants were analyzed using liquid chromatography - high-resolution mass spectrometry (LC-HRMS) method as previously described by Teav *et al* ^12^.

## **Bioinformatics analysis**

All code has been run using the open-source programming language, Python 3.9.5 ^13^, all processing of the data has been done using the Pandas package (v1.2.4),^14^ and figures were generated with the Plotly library (v5.5.0) ^15^. The scikit-learn library (v0.24.2) ^16^ was used for normalization with the function StandardScaler, subtracting the mean from each sample and dividing by the standard deviation. Principal components were obtained with the function PCA from the same library. For the clinical PCA, missing values were filled with the mean of the group. Pearson correlations were computed with Scipy (v1.6.2) ^17^ and used to (i) evaluate the contribution of individual variables to specific PCs after PCA, and (ii) correlate patient phenotypes with gene expression. For correlations with omics data, p values were adjusted using Benjamini-Hochberg false discovery rate (FDR) method, while for analysis with basic phenotypic data we used Bonferroni multiple test correction based on categories of independent variables: **Age** (Calculated age); **Anthropometrics** (Height, BMI [kg/m2], Android/Gynoid ratio, ALMI, Lean mass index); **Metabolic comorbidities** (Metabolic syndrome ATP-III criteria [max 5], Arterial pressure sys, Arterial pressure dia, Arterial Hypertension, OSAS; **Glucose Metabolism** (Glucose, Insulin, HOMA-IR); **Adiposity-Adipokines** (Fat percent, Intra-visceral fat mass, Fat mass index, Leptin AM [ng/ml]); **Lipids** (Cholesterol, HDL, Triglycerides, LDL calculated); **Liver Steatosis** (ALAT, NAFLD score); **Stress and inflammation** (hs-CRP, Cortisol AM [nmol/L]); **Reproductive status** (T/E, Testosterone AM [nmol/l], Free T [pmol/l], SHBG [nmol/l], LH [U/l], FSH [U/l], Estradiol [nmol/l]); **Metabolic regulators** (FGF21 AM, TSH, FGF19 AM).

## **Sensitivity analysis**

Comparisons of the main descriptive parameters between the two obese groups were adjusted for age or BMI to account for a potentially confounding effect (Table S2). For this analysis, the difference between the mean of the groups for continuous variables and the odds ratio for categorical variables was assessed by applying linear and logistic regressions, respectively.

# **SUPPLEMENTARY FIGURES**

## **Figure S1. Flow chart of the study**


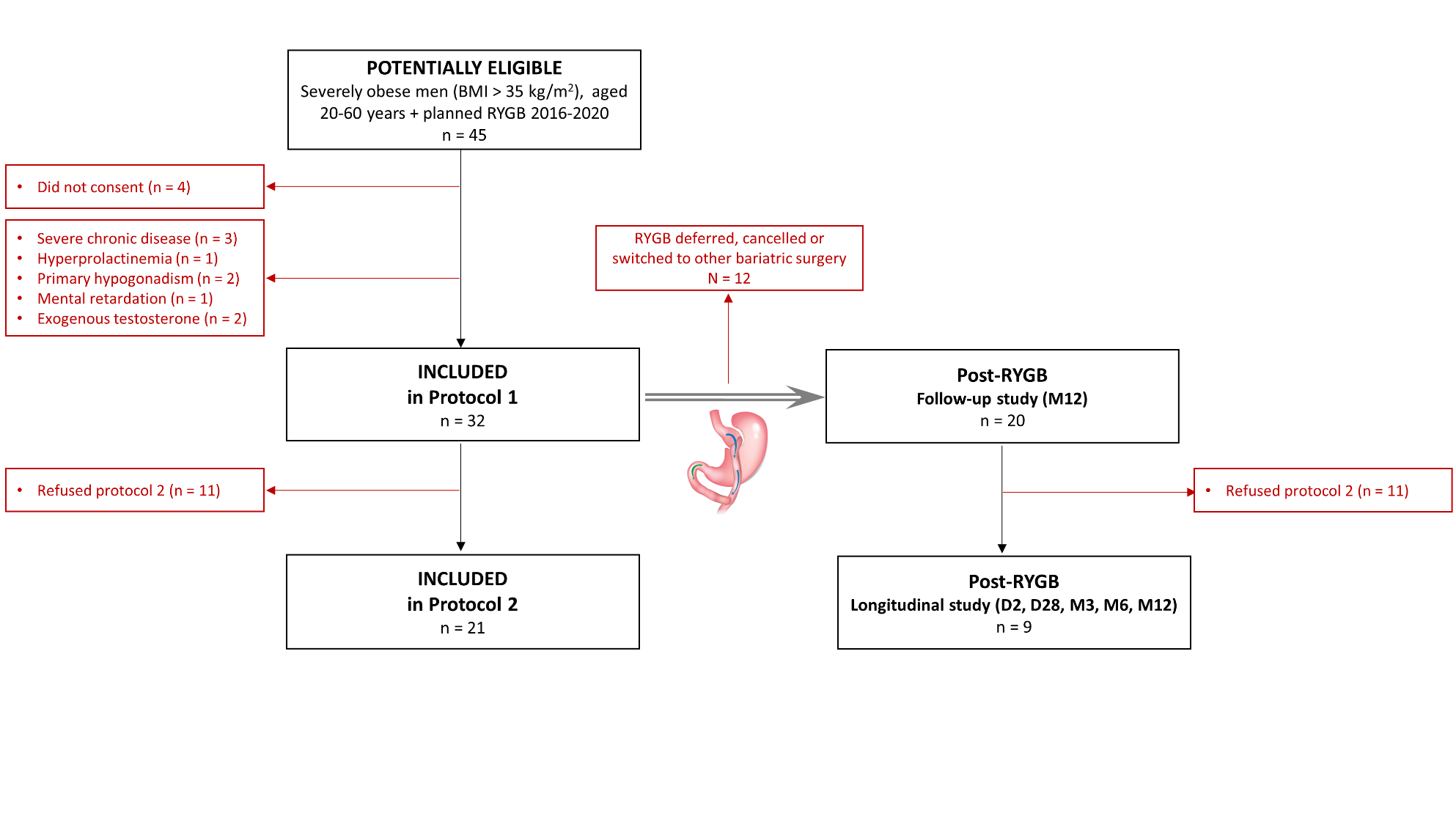


## **Figure S2. Free testosterone by calculation versus direct measurement and impact on the classification of the obese cohort**

FT-V, free testosterone calculated with the Vermeulen formula ^6^; FT-ED, free testosterone directly measured by equilibrium dialysis. ObHH, obese with hypogonadotropic hypogonadism, shown in red circles; ObnHH, obese without hypogonadotropic hypogonadism, shown in blue squares; Lean controls, shown in green triangles. In panel B, two subjects in ObHH (shown in blue rectangles) and one subject in ObnHH (shown in red circle) would have switched groups if the alternative cut-off of FT-V at 220 pmol/l, illustrated by the dotted line, would be used to define HH.

## **Figure S3. Glucose and insulin changes following a standard 2h oral glucose tolerance test**

OGTT, oral glucose tolerance test. ObHH, obese with hypogonadotropic hypogonadism. ObnHH, obese without hypogonadotropic hypogonadism

* p<0.05 as compared to ObnHH and Lean; ** p<0.01 as compared to ObnHH and Lean

## **Figure S4: Linear regression of metabolic parameters with testosterone, SHBG and free testosterone in the obese cohort**

SHBG, sex-hormone binding globulin; HOMA-IR, homeostatic assessment insulin resistance index; hs-CRP, high-sensitivity C-reactive protein

ObHH, obese with hypogonadotropic hypogonadism are shown in red.

ObnHH, obese without hypogonadotropic hypogonadism are shown in blue.

Significant associations are highlighted in bold.

## **Figure S5. Metabolic phenotype according to obesity and reproductive status**

ObHH, obese with hypogonadotropic hypogonadism. ObnHH, obese without hypogonadotropic hypogonadism

**** p<0.0001; *** p<0.001; ns, not significant

## **Figure S6: Linear Regression of insulin resistance index and serum leptin levels with fractional anisotropy**

HOMA-IR : Homeostatic model assessment for insulin resistance. Fractional Anisotropy was derived from diffusion-tensor imaging MRI.

## **Figure S7: Total and specific fatty acyl chain length ceramides and other sphingolipids**

Plasma levels of principal classes of sphingolipids as measured by liquid chromatography – tandem mass spectrometry (LC-MS/MS, see chapter 2.4 in Supplementary Methods). The number following the letter C indicates the length of the fatty acyl chain, which is a crucial element for their metabolic effect ^18^. DhCer, Dihydroceramides; ObHH, obese with hypogonadotropic hypogonadism; ObnHH, obese without hypogonadotropic hypogonadism. * p< 0.05; ** p< 0.01; *** p<0.001; **** p<0.0001; ns, not significant

## **Figure S8: Linear regression of ceramides and selected dihydroceramides and deoxysphingolipids with insulin resistance index, free testosterone levels and FGF21**

HOMA-IR : Homeostatic model assessment for insulin resistance, FT: Free testosterone, DhCer: Dihydroceramides, 1-Deoxy-Spa (m18:0/16:0), 1-deoxysphinganine (m18:0). Plasma levels of principal classes of sphingolipids as measured by liquid chromatography – tandem mass spectrometry (LC-MS/MS, see chapter 2.4 in Supplementary Methods).

## **Figure S9: Functional enrichment analysis of GPC.2 correlating genes related signaling pathways**


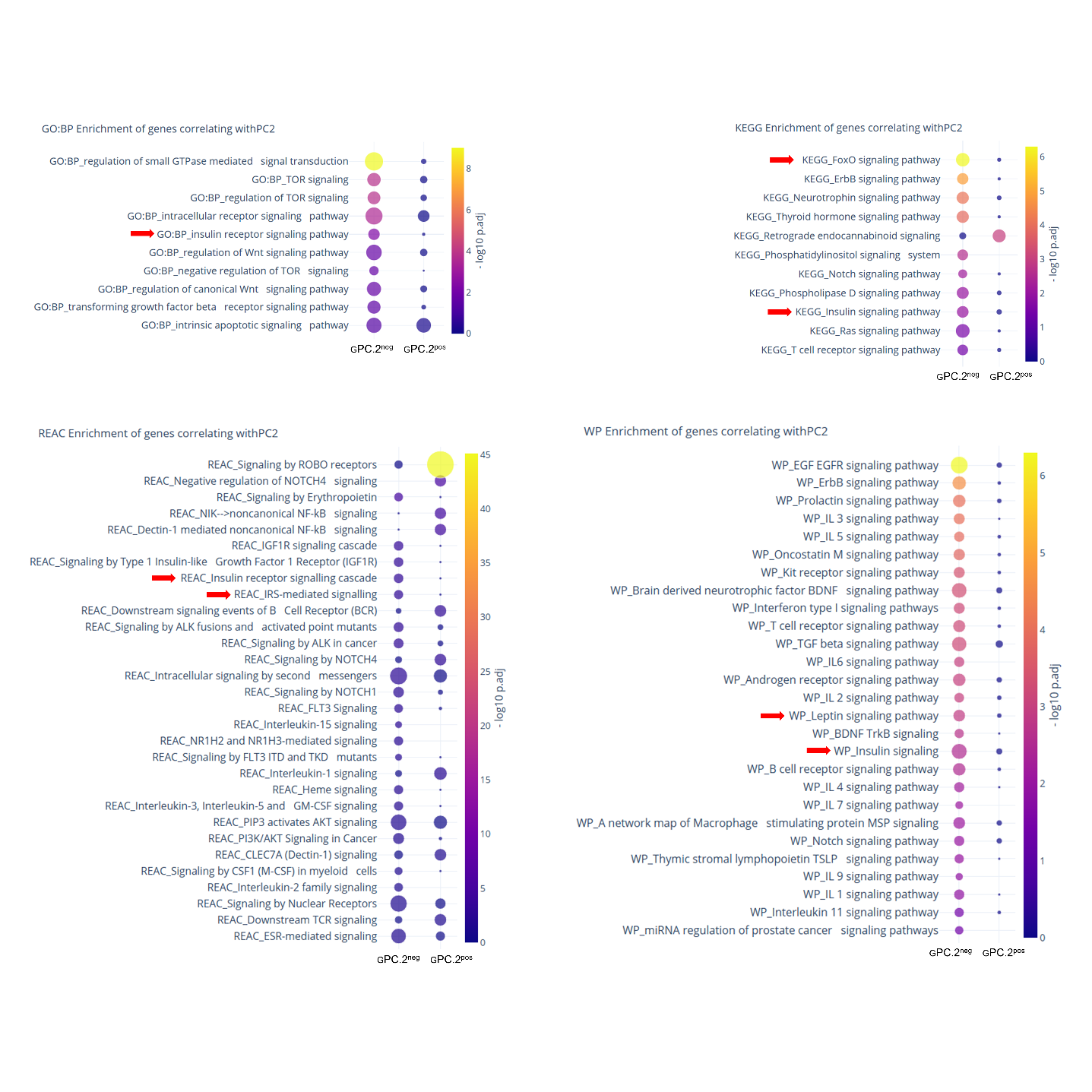


PC.2 correlating genes have been selected based on correlation significance (FDR 5%). Enrichment of positively (GPC2pos) and negatively (GPC.2neg) genes has been calculated using (A) GO:BP, (B) KEGG, (C) Reactome, and (D) WikiPathways databases. Plots represent all significant enriched terms after filtering (Term name: “signaling”; Term size: < 400). Red arrows highlight pathways linked with insulin and leptin signaling.

## **Figure S10: Change in fractional anisotropy in six patients with available diffusion-tensor Imaging MRI at baseline and month 12 post RYGB**

RYGB, Roux-en-Y Gastric Bypass; M12, Month 12; ns, not significant.

## **Figure S11: Longitudinal changes post RYGB in key metabolic and reproductive outcomes**

Post-RYGB changes using linear mixed model regression in nine subjects that consented to a longitudinal follow-up with multiple visits. Data are shown as mean ± standard error. SHBG, sex-hormone binding globulin. * p<0.05; ** p<0.01; *** p<0.001; **** p<0.0001

## **Figure S12: Longitudinal changes post RYGB in fasting plasma levels of branched-chain amino acids**

Data are shown as mean ± standard deviation. RYGB, Roux-en-Y Gastric Bypass. Given the suspected link of amino-acid restriction with FGF21 induction, occurring in our cohort at Day 28, the differences of amino-acids using linear mixed model regression in comparison to baseline are visualized. * p<0.05; ** p<0.001; *** p<0.001; **** p<0.001; ns, not significant.

## **Figure S13: Change in metabolic markers as potential predictors of the rise of serum T levels at month 12 post RYGB**

Linear regression of the delta (Δ) changes in different metabolic parameters vs the delta changes of serum testosterone levels at Month 12 (M12) post Roux-en-Y Gastric Bypass (RYGB). Obese with and without hypogonadotropic hypogonadism are shown in red (ObHH) and blue (ObnHH). BMI, body mass index; HOMA-IR, homeostatic assessment insulin resistance index; D28, day 28 post RYGB; M12, month 12 post RYGB; FM, fat mass; hs-CRP, high-sensitivity C-reactive protein. With the exception of FGF21 and leptin changes (see Main Figure 4), no other parameter significantly predicted the extent of HH reversal.

# **SUPPLEMENTARY TABLES**

## **Table S1: Differences of obese groups in baseline characteristics after adjusting for age or body mass index**

|  | BASELINE DIFFERENCES (ObHH vs ObnHH) | | | | |  |
| --- | --- | --- | --- | --- | --- | --- |
|  | No adjustment | p-value | Adjustment for age | p-value | Adjustment for BMI | p-value |
| *Metabolic comorbidities* |  |  |  |  |  |  |
| Metabolic syndrome (yes/no) | 5.8 (1.1, 44.7) | 0.05 | 8.4 (1.1, 158.9) | 0.08 | 5.4 (1, 42) | 0.06 |
| Hypertension (yes/no) | 11.9 (2.3, 94.7) | **0.007** | 11.4 (2, 104.4) | **0.01** | 17.6 (2.9, 213) | **0.006** |
| *Glucose metabolism* |  |  |  |  |  |  |
| Glucose (mmol/l) | 1.0 (0.3, 1.7) | **0.008** | 0.8 (0.1, 1.6) | **0.02** | 1.1 (0.3, 1.8) | **0.006** |
| Insulin (mU/l) | 26.9 (11.8, 42) | **0.001** | 25.2 (9.9, 40.5) | **0.002** | 25.6 (9.6, 41.5) | **0.003** |
| HOMA-IR | 8.7 (4.2, 13.3) | **0.0005** | 8.1 (3.6, 12.7) | **0.001** | 8.4 (3.6, 13.2) | **0.001** |
| Hb1Ac % | 0.8 (0.1, 1.5) | **0.03** | 0.7 (0.001, 1.4) | **0.05** | 0.8 (0.2, 1.5) | **0.02** |
| *Adiposity-Adipokines* |  |  |  |  |  |  |
| Leptin (ng/ml) | 15.3 (-9.1, 39.8) | 0.21 | 17.5 (-7.8, 42.8) | 0.17 | 7.2 (-14.1, 28.6) | 0.49 |
| Fat mass percentage (%) | 1.4 (-2.7, 5.6) | 0.48 | 1.9 (-2.2, 6.1) | 0.35 | -0.8 (-2.9, 1.2) | 0.35 |
| Visceral adipose tissue (g) | 850 (-135, 1835) | 0.09 | 843 (-175, 1862) | 0.10 | 519 (-346, 1385) | 0.23 |
| *Lipids* |  |  |  |  |  |  |
| Total cholesterol (mmol/l) | -0.2 (-0.7, 0.3) | 0.41 | -0.3 (-0.9, 0.2) | 0.25 | -0.2 (-0.7, 0.4) | 0.55 |
| Triglycerides (mmol/l) | 0.7 (0.1, 1.4) | **0.02** | 0.7 (0.01, 1.3) | **0.05** | 0.8 (0.1, 1.4) | **0.01** |
| HDL cholesterol (mmol/l) | -0.25 (-0.41, -0.10) | **0.002** | -0.27 (-0.43, -0.10) | **0.002** | -0.24 (-0.40, -0.08) | **0.004** |
| *Liver steatosis* |  |  |  |  |  |  |
| ALAT (IU/l) | -6 (-21, 8) | 0.37 | -6 (-21, 9) | 0.41 | -6 (-21, 9) | 0.39 |
| ASAT (IU/l) | -7 (-13, 0.2) | 0.06 | -6 (-13, 1.0) | 0.09 | -6 (-13, 0.7) | 0.09 |
| NAFLD Fibrosis Score | 0.20 (-0.67, 1.08) | 0.63 | 0.07 (-0.81, 0.95) | 0.87 | 0.06 (-0.78, 0.89) | 0.89 |
| *Stress and inflammation* |  |  |  |  |  |  |
| hsCRP (mg/l) | 2.8 (-0.5, 6.1) | 0.09 | 3.6 (0.5, 6.8) | **0.02** | 1.8 (-1.2, 4.8) | 0.23 |
| Cortisol (nmol/l) | 12 (-57, 82) | 0.72 | 18 (-54, 90) | 0.61 | 8 (-63, 79) | 0.81 |
| Reproductive status |  |  |  |  |  |  |
| ADAM score (positive/negative) | 2.5 (0.4, 21.9) | 0.40 | 4.3 (0.4, 102) | 0.26 | 256 (3.2, 327768) | 0.05 |
| Total testosterone (nmol/l) | -7.0 (-8.7, -5.4) | **<0.0001** | -7.4 (-9.0, -5.7) | **<0.0001** | -6.8 (-8.4, -5.2) | **<0.0001** |
| Free testosterone (pmol/l) | -122 (-155, -89) | **<0.0001** | -121 (-155, -86) | **<0.0001** | -117 (-148, -85) | **<0.0001** |
| SHBG (nmol/l) | -9.7 (-19.2, -0.2) | **0.05** | -11.2 (-20.8, -1.5) | **0.02** | -10.2 (-20, -0.5) | **0.04** |
| LH (IU/l) | 0.1 (-1.6, 1.7) | 0.95 | -0.3 (-2.0, 1.3) | 0.70 | 0.1 (-1.6, 1.9) | 0.87 |
| FSH (IU/l) | 1.0 (-1.4, 3.3) | 0.40 | 0.4 (-1.8, 2.6) | 0.74 | 1.1 (-1.2, 3.5) | 0.34 |
| Estradiol (nmol/l) | -0.00 (-0.04, 0.03) | 0.84 | 0.01 (-0.02, 0.04) | 0.69 | -0.01 (-0.04, 0.02) | 0.44 |
| Testo/Estradiol ratio | -54 (-80, -29) | **0.0001** | -60 (-85, -36) | **<0.0001** | -49 (-71, -26) | **0.0001** |

Metabolic syndrome was defined based on the ATP-III criteria ^4^. NAFLD (non-alcoholic fatty liver disease) fibrosis score was calculated based on a published algorithm ^5^. Data are shown as estimated differences in mean levels except for the first two categorical variable for which odds ratio are given. Low and high confidence interval limits are displayed in parenthesis. Statistically significant differences (p<0.05) are shown in bold.

|  | **n** | **P-value (univar)** | **beta** | **2.5 %** | **97.5 %** | **P-value (multivar)** |
| --- | --- | --- | --- | --- | --- | --- |
| **Total Testosterone** |  |  |  |  |  |  |
| (Intercept) | 26 | - | 14.53854324 | 2.542379319 | 26.53470715 | 0.02 |
| Age | 26 | 0.98 | 0.067476207 | -0.176331574 | 0.311283987 | 0.57 |
| HOMA-IR | 26 | **0.008** | -0.270512416 | -0.523389882 | -0.017634949 | **0.04** |
| hs-CRP | 26 | 0.11 | -0.157316124 | -0.529009714 | 0.214377467 | 0.39 |
| Visceral Adipose Tissue | 26 | 0.05 | -0.000599167 | -0.001886517 | 0.000688183 | 0.34 |
| **Free Testosterone** |  |  |  |  |  |  |
| (Intercept) | 26 |  | 355.4483381 | 154.6202827 | 556.2763936 | 0.001390264 |
| Age | 26 | 0.57 | 0.349085673 | -3.732505979 | 4.430677324 | 0.86 |
| HOMA-IR | 26 | **0.0008** | -5.676668598 | -9.910096072 | -1.443241123 | **0.01** |
| hs-CRP | 26 | 0.12 | -2.849828933 | -9.072359861 | 3.372701995 | 0.35 |
| Visceral Adipose Tissue | 26 | **0.02** | -0.012906845 | -0.034458398 | 0.008644708 | 0.23 |

## **Table S2: Multivariate analysis of the effect of metabolic parameters on total and free testosterone of obese men**

Statistically significant differences (p<0.05) are shown in bold.

## **Table S3: Genes drivers of principal component 2 of blood transcriptomics**

| **#** | **Ensembl ID** | **Symbol** | **r** | **p value** | **Gene Name/Gene Symbol** | **PANTHER Family/Subfamily** |
| --- | --- | --- | --- | --- | --- | --- |
| **1** | ENSG00000100632 | ERH | 0.95 | 1.95E-13 | Enhancer of rudimentary homolog;ERH;ortholog | ENHANCER OF RUDIMENTARY HOMOLOG (PTHR12373:SF9) |
| **2** | ENSG00000169442 | HEL-S-171mP | 0.95 | 2.04E-13 | CAMPATH-1 antigen;CD52;ortholog | CAMPATH-1 ANTIGEN (PTHR15029:SF0) |
| **3** | ENSG00000198918 | RPL39 | 0.95 | 2.05E-13 | 60S ribosomal protein L39;RPL39;ortholog | 60S RIBOSOMAL PROTEIN L39 (PTHR19970:SF25) |
| **4** | ENSG00000189043 | NDUFA4 | 0.94 | 5.32E-13 | Cytochrome c oxidase subunit NDUFA4;NDUFA4;ortholog | CYTOCHROME C OXIDASE SUBUNIT NDUFA4 (PTHR14256:SF4) |
| **5** | ENSG00000127184 | COX7C | 0.93 | 9.74E-12 | Cytochrome c oxidase subunit 7C, mitochondrial;COX7C;ortholog | CYTOCHROME C OXIDASE SUBUNIT 7C, MITOCHONDRIAL (PTHR13313:SF1) |
| **6** | ENSG00000136942 | RPL35 | 0.93 | 5.07E-12 | 60S ribosomal protein L35;RPL35;ortholog | 60S RIBOSOMAL PROTEIN L35 (PTHR45722:SF9) |
| **7** | ENSG00000139168 | ZCRB1 | 0.93 | 1.14E-11 | Zinc finger CCHC-type and RNA-binding motif-containing protein 1;ZCRB1;ortholog | ZINC FINGER CCHC-TYPE AND RNA-BINDING MOTIF-CONTAINING PROTEIN 1 (PTHR46259:SF2) |
| **8** | ENSG00000140319 | SRP14 | 0.93 | 1.26E-11 | Signal recognition particle 14 kDa protein;SRP14;ortholog | SIGNAL RECOGNITION PARTICLE 14 KDA PROTEIN (PTHR12013:SF2) |
| **9** | ENSG00000161057 | PSMC2 | 0.93 | 6.88E-12 | 26S proteasome regulatory subunit 7;PSMC2;ortholog | 26S PROTEASOME REGULATORY SUBUNIT 7 (PTHR23073:SF13) |
| **10** | ENSG00000110200 | ANAPC15 | 0.92 | 4.92E-11 | Anaphase-promoting complex subunit 15;ANAPC15;ortholog | ANAPHASE-PROMOTING COMPLEX SUBUNIT 15 (PTHR22526:SF4) |
| **11** | ENSG00000112695 | COX7A2 | 0.92 | 2.81E-11 |  |  |
| **12** | ENSG00000114391 | RPL24 | 0.92 | 5.17E-11 | 60S ribosomal protein L24;RPL24;ortholog | 60S RIBOSOMAL PROTEIN L24 (PTHR10792:SF44) |
| **13** | ENSG00000140612 | SEC11A | 0.92 | 1.94E-11 | Signal peptidase complex catalytic subunit SEC11A;SEC11A;ortholog | SIGNAL PEPTIDASE COMPLEX CATALYTIC SUBUNIT SEC11A (PTHR10806:SF36) |
| **14** | ENSG00000169567 | HINT1 | 0.92 | 3.94E-11 | Histidine triad nucleotide-binding protein 1;HINT1;ortholog | HISTIDINE TRIAD NUCLEOTIDE-BINDING PROTEIN 1 (PTHR23089:SF45) |
| **15** | ENSG00000171863 | RPS7 | 0.92 | 5.87E-11 | 40S ribosomal protein S7;RPS7;ortholog | 40S RIBOSOMAL PROTEIN S7 (PTHR11278:SF5) |
| **16** | ENSG00000173660 | UQCRH | 0.92 | 1.84E-11 | Cytochrome b-c1 complex subunit 6, mitochondrial;UQCRH;ortholog | CYTOCHROME B-C1 COMPLEX SUBUNIT 6, MITOCHONDRIAL (PTHR15336:SF3) |
| **17** | ENSG00000174547 | MRPL11 | 0.92 | 3.67E-11 | 39S ribosomal protein L11, mitochondrial;MRPL11;ortholog | 39S RIBOSOMAL PROTEIN L11, MITOCHONDRIAL (PTHR11661:SF19) |
| **18** | ENSG00000177954 | RPS27 | 0.92 | 1.82E-11 | 40S ribosomal protein S27;RPS27;ortholog | 40S RIBOSOMAL PROTEIN S27 (PTHR11594:SF1) |
| **19** | ENSG00000182899 | RPL35A | 0.92 | 4.51E-11 | 60S ribosomal protein L35a;RPL35A;ortholog | 60S RIBOSOMAL PROTEIN L35A (PTHR10902:SF30) |
| **20** | ENSG00000240889 | NDUFB2-AS1 | 0.92 | 4.10E-11 |  |  |
| **21** | ENSG00000041357 | PSMA4 | 0.91 | 2.03E-10 | Proteasome subunit alpha type-4;PSMA4;ortholog | PROTEASOME SUBUNIT ALPHA TYPE-4 (PTHR11599:SF13) |
| **22** | ENSG00000111832 | RWDD1 | 0.91 | 1.76E-10 | RWD domain-containing protein 1;RWDD1;ortholog | RWD DOMAIN-CONTAINING PROTEIN 1 (PTHR12292:SF2) |
| **23** | ENSG00000123349 | PFDN5 | 0.91 | 6.28E-11 | Prefoldin subunit 5;PFDN5;ortholog | PREFOLDIN SUBUNIT 5 (PTHR12674:SF6) |
| **24** | ENSG00000125691 | RPL23 | 0.91 | 1.54E-10 | 60S ribosomal protein L23;RPL23;ortholog | 60S RIBOSOMAL PROTEIN L23 (PTHR11761:SF22) |
| **25** | ENSG00000125870 | SNRPB2 | 0.91 | 9.16E-11 | U2 small nuclear ribonucleoprotein B'';SNRPB2;ortholog | U2 SMALL NUCLEAR RIBONUCLEOPROTEIN B'' (PTHR10501:SF84) |
| **26** | ENSG00000131469 | RPL27 | 0.91 | 1.39E-10 | 60S ribosomal protein L27;RPL27;ortholog | 60S RIBOSOMAL PROTEIN L27 (PTHR10497:SF11) |
| **27** | ENSG00000137154 | RPS6 | 0.91 | 1.20E-10 | 40S ribosomal protein S6;RPS6;ortholog | 40S RIBOSOMAL PROTEIN S6 (PTHR11502:SF34) |
| **28** | ENSG00000164919 | COX6C | 0.91 | 1.33E-10 |  |  |
| **29** | ENSG00000184076 | UQCR10 | 0.91 | 1.86E-10 | Cytochrome b-c1 complex subunit 9;UQCR10;ortholog | CYTOCHROME B-C1 COMPLEX SUBUNIT 9 (PTHR12980:SF0) |
| **30** | ENSG00000232112 | TMA7 | 0.91 | 8.93E-11 | Translation machinery-associated protein 7;TMA7;ortholog | TRANSLATION MACHINERY-ASSOCIATED PROTEIN 7 (PTHR28632:SF11) |
| **31** | ENSG00000008018 | PSMB1 | 0.9 | 2.77E-10 | Proteasome subunit beta type-1;PSMB1;ortholog | PROTEASOME SUBUNIT BETA TYPE-1 (PTHR32194:SF2) |
| **32** | ENSG00000071082 | RPL31 | 0.9 | 3.26E-10 | 60S ribosomal protein L31;RPL31;ortholog | 60S RIBOSOMAL PROTEIN L31 (PTHR10956:SF4) |
| **33** | ENSG00000115128 | SF3B6 | 0.9 | 6.37E-10 | Splicing factor 3B subunit 6;SF3B6;ortholog | SPLICING FACTOR 3B SUBUNIT 6 (PTHR12785:SF7) |
| **34** | ENSG00000116459 | ATP5PB | 0.9 | 2.55E-10 | ATP synthase F(0) complex subunit B1, mitochondrial;ATP5PB;ortholog | ATP SYNTHASE F(0) COMPLEX SUBUNIT B1, MITOCHONDRIAL (PTHR12733:SF3) |
| **35** | ENSG00000118640 | VAMP8 | 0.9 | 2.91E-10 | Vesicle-associated membrane protein 8;VAMP8;ortholog | VESICLE-ASSOCIATED MEMBRANE PROTEIN 8 (PTHR45701:SF7) |
| **36** | ENSG00000125743 | SNRPD2 | 0.9 | 3.03E-10 | Small nuclear ribonucleoprotein Sm D2;SNRPD2;ortholog | SMALL NUCLEAR RIBONUCLEOPROTEIN SM D2 (PTHR12777:SF0) |
| **37** | ENSG00000131174 | COX7B | 0.9 | 3.39E-10 | Cytochrome c oxidase subunit 7B, mitochondrial;COX7B;ortholog | CYTOCHROME C OXIDASE SUBUNIT 7B, MITOCHONDRIAL (PTHR16716:SF0) |
| **38** | ENSG00000138382 | METTL5 | 0.9 | 2.66E-10 | rRNA N6-adenosine-methyltransferase METTL5;METTL5;ortholog | RRNA N6-ADENOSINE-METHYLTRANSFERASE METTL5 (PTHR23290:SF0) |
| **39** | ENSG00000143947 | RPS27A | 0.9 | 5.43E-10 | Ubiquitin-40S ribosomal protein S27a;RPS27A;ortholog | UBIQUITIN-40S RIBOSOMAL PROTEIN S27A (PTHR10666:SF442) |
| **40** | ENSG00000165629 | ATP5F1C | 0.9 | 3.55E-10 | ATP synthase subunit gamma, mitochondrial;ATP5F1C;ortholog | ATP SYNTHASE SUBUNIT GAMMA, MITOCHONDRIAL (PTHR11693:SF31) |
| **41** | ENSG00000166562 | SEC11C | 0.9 | 3.15E-10 | Signal peptidase complex catalytic subunit SEC11C;SEC11C;ortholog | SIGNAL PEPTIDASE COMPLEX CATALYTIC SUBUNIT SEC11C (PTHR10806:SF12) |
| **42** | ENSG00000229117 | RPL41 | 0.9 | 4.89E-10 |  |  |
| **43** | ENSG00000231500 | RPS18 | 0.9 | 2.53E-10 | 40S ribosomal protein S18;RPS18;ortholog | 40S RIBOSOMAL PROTEIN S18 (PTHR10871:SF42) |
| **44** | ENSG00000000419 | DPM1 | 0.89 | 1.12E-09 | Dolichol-phosphate mannosyltransferase subunit 1;DPM1;ortholog | DOLICHOL-PHOSPHATE MANNOSYLTRANSFERASE SUBUNIT 1 (PTHR43398:SF1) |
| **45** | ENSG00000092841 | MYL6 | 0.89 | 1.79E-09 | Myosin light polypeptide 6;MYL6;ortholog | MYOSIN LIGHT POLYPEPTIDE 6 (PTHR23048:SF43) |
| **46** | ENSG00000116030 | SUMO1 | 0.89 | 7.72E-10 | Small ubiquitin-related modifier 1;SUMO1;ortholog | SMALL UBIQUITIN-RELATED MODIFIER 1 (PTHR10562:SF117) |
| **47** | ENSG00000132341 | RAN | 0.89 | 1.93E-09 | GTP-binding nuclear protein Ran;RAN;ortholog | GTP-BINDING NUCLEAR PROTEIN RAN (PTHR24071:SF37) |
| **48** | ENSG00000134597 | RBMX2 | 0.89 | 7.72E-10 | RNA-binding motif protein, X-linked 2;RBMX2;ortholog | RNA-BINDING MOTIF PROTEIN, X-LINKED 2 (PTHR45880:SF4) |
| **49** | ENSG00000142168 | SOD1 | 0.89 | 1.12E-09 | Superoxide dismutase [Cu-Zn];SOD1;ortholog | SUPEROXIDE DISMUTASE [CU-ZN] (PTHR10003:SF71) |
| **50** | ENSG00000143183 | TMCO1 | 0.89 | 1.58E-09 | Calcium load-activated calcium channel;TMCO1;ortholog | CALCIUM LOAD-ACTIVATED CALCIUM CHANNEL (PTHR20917:SF0) |
| **51** | ENSG00000144034 | TPRKB | 0.89 | 9.92E-10 | EKC/KEOPS complex subunit TPRKB;TPRKB;ortholog | EKC/KEOPS COMPLEX SUBUNIT TPRKB (PTHR15840:SF10) |
| **52** | ENSG00000147687 | TATDN1 | 0.89 | 1.16E-09 | Putative deoxyribonuclease TATDN1;TATDN1;ortholog | DEOXYRIBONUCLEASE TATDN1-RELATED (PTHR10060:SF15) |
| **53** | ENSG00000155368 | DBI | 0.89 | 1.36E-09 | Acyl-CoA-binding protein;DBI;ortholog | ACYL-COA-BINDING PROTEIN (PTHR23310:SF54) |
| **54** | ENSG00000158062 | UBXN11 | 0.89 | 7.68E-10 | UBX domain-containing protein 11;UBXN11;ortholog | UBX DOMAIN-CONTAINING PROTEIN 11 (PTHR23333:SF4) |
| **55** | ENSG00000196262 | HEL-S-69p | 0.89 | 7.25E-10 | Peptidyl-prolyl cis-trans isomerase A;PPIA;ortholog | PEPTIDYL-PROLYL CIS-TRANS ISOMERASE A (PTHR11071:SF490) |
| **56** | ENSG00000077380 | DYNC1I2 | 0.88 | 3.52E-09 | Cytoplasmic dynein 1 intermediate chain 2;DYNC1I2;ortholog | CYTOPLASMIC DYNEIN 1 INTERMEDIATE CHAIN 2 (PTHR12442:SF37) |
| **57** | ENSG00000080824 | EL52 | 0.88 | 2.74E-09 | Heat shock protein HSP 90-alpha;HSP90AA1;ortholog | HEAT SHOCK PROTEIN HSP 90-ALPHA (PTHR11528:SF87) |
| **58** | ENSG00000089009 | RPL6 | 0.88 | 4.27E-09 | 60S ribosomal protein L6;RPL6;ortholog | 60S RIBOSOMAL PROTEIN L6 (PTHR10715:SF7) |
| **59** | ENSG00000089157 | RPLP0 | 0.88 | 3.65E-09 | 60S acidic ribosomal protein P0;RPLP0;ortholog | 60S ACIDIC RIBOSOMAL PROTEIN P0-RELATED (PTHR45699:SF1) |
| **60** | ENSG00000114942 | EEF1B2 | 0.88 | 3.79E-09 | Elongation factor 1-beta;EEF1B2;ortholog | ELONGATION FACTOR 1-BETA (PTHR11595:SF60) |
| **61** | ENSG00000125356 | ZNF183 | 0.88 | 2.32E-09 | NADH dehydrogenase [ubiquinone] 1 alpha subcomplex subunit 1;NDUFA1;ortholog | NADH DEHYDROGENASE [UBIQUINONE] 1 ALPHA SUBCOMPLEX SUBUNIT 1 (PTHR17098:SF2) |
| **62** | ENSG00000125977 | EIF2S2 | 0.88 | 4.78E-09 | Eukaryotic translation initiation factor 2 subunit 2;EIF2S2;ortholog | EUKARYOTIC TRANSLATION INITIATION FACTOR 2 SUBUNIT 2 (PTHR23001:SF24) |
| **63** | ENSG00000143420 | ENSA | 0.88 | 4.07E-09 | Alpha-endosulfine;ENSA;ortholog | ALPHA-ENDOSULFINE (PTHR10358:SF21) |
| **64** | ENSG00000147604 | RPL7 | 0.88 | 4.13E-09 | 60S ribosomal protein L7;RPL7;ortholog | 60S RIBOSOMAL PROTEIN L7 (PTHR11524:SF12) |
| **65** | ENSG00000164405 | UQCRQ | 0.88 | 2.04E-09 | Cytochrome b-c1 complex subunit 8;UQCRQ;ortholog | CYTOCHROME B-C1 COMPLEX SUBUNIT 8 (PTHR12119:SF2) |
| **66** | ENSG00000171530 | TBCA | 0.88 | 4.50E-09 | Tubulin-specific chaperone A;TBCA;ortholog | TUBULIN-SPECIFIC CHAPERONE A (PTHR21500:SF3) |
| **67** | ENSG00000172172 | MRPL13 | 0.88 | 4.82E-09 | 39S ribosomal protein L13, mitochondrial;MRPL13;ortholog | 39S RIBOSOMAL PROTEIN L13, MITOCHONDRIAL (PTHR11545:SF2) |
| **68** | ENSG00000174720 | LARP7 | 0.88 | 3.55E-09 | La-related protein 7;LARP7;ortholog | LA-RELATED PROTEIN 7 (PTHR22792:SF115) |
| **69** | ENSG00000198258 | UBL5 | 0.88 | 1.94E-09 | Ubiquitin-like protein 5;UBL5;ortholog | UBIQUITIN-LIKE PROTEIN 5 (PTHR13042:SF3) |
| **70** | ENSG00000249532 | MIR302CHG | 0.88 | 3.33E-09 |  |  |
| **71** | ENSG00000034510 | TMSB10 | 0.87 | 5.37E-09 | Thymosin beta-10;TMSB10;ortholog | THYMOSIN BETA-10 (PTHR12021:SF10) |
| **72** | ENSG00000078668 | VDAC3 | 0.87 | 7.24E-09 | Voltage-dependent anion-selective channel protein 3;VDAC3;ortholog | VOLTAGE-DEPENDENT ANION-SELECTIVE CHANNEL PROTEIN 3 (PTHR11743:SF28) |
| **73** | ENSG00000087302 | C14orf166 | 0.87 | 9.85E-09 | RNA transcription, translation and transport factor protein;RTRAF;ortholog | RNA TRANSCRIPTION, TRANSLATION AND TRANSPORT FACTOR PROTEIN (PTHR15924:SF11) |
| **74** | ENSG00000102309 | PIN4 | 0.87 | 8.68E-09 | Peptidyl-prolyl cis-trans isomerase NIMA-interacting 4;PIN4;ortholog | PEPTIDYL-PROLYL CIS-TRANS ISOMERASE NIMA-INTERACTING 4 (PTHR45995:SF2) |
| **75** | ENSG00000109083 | IFT20 | 0.87 | 8.40E-09 | Intraflagellar transport protein 20 homolog;IFT20;ortholog | INTRAFLAGELLAR TRANSPORT PROTEIN 20 HOMOLOG (PTHR31978:SF1) |
| **76** | ENSG00000117133 | RPF1 | 0.87 | 1.00E-08 | Ribosome production factor 1;RPF1;ortholog | RIBOSOME PRODUCTION FACTOR 1 (PTHR22734:SF3) |
| **77** | ENSG00000117450 | PRDX1 | 0.87 | 1.18E-08 | Peroxiredoxin-1;PRDX1;ortholog | PEROXIREDOXIN-1 (PTHR10681:SF111) |
| **78** | ENSG00000122026 | RPL21 | 0.87 | 6.31E-09 | 60S ribosomal protein L21;RPL21;ortholog | 60S RIBOSOMAL PROTEIN L21 (PTHR20981:SF8) |
| **79** | ENSG00000134375 | TIMM17A | 0.87 | 1.08E-08 | Mitochondrial import inner membrane translocase subunit Tim17-A;TIMM17A;ortholog | MITOCHONDRIAL IMPORT INNER MEMBRANE TRANSLOCASE SUBUNIT TIM17-A (PTHR10485:SF1) |
| **80** | ENSG00000142676 | RPL11 | 0.87 | 1.03E-08 | 60S ribosomal protein L11;RPL11;ortholog | 60S RIBOSOMAL PROTEIN L11 (PTHR11994:SF23) |
| **81** | ENSG00000156411 | ATP5MPL | 0.87 | 6.90E-09 | ATP synthase subunit ATP5MJ, mitochondrial;ATP5MJ;ortholog | ATP SYNTHASE SUBUNIT ATP5MJ, MITOCHONDRIAL (PTHR15233:SF1) |
| **82** | ENSG00000163682 | RPL9 | 0.87 | 8.69E-09 | 60S ribosomal protein L9;RPL9P9;ortholog | 60S RIBOSOMAL PROTEIN L9 (PTHR11655:SF46) |
| **83** | ENSG00000164587 | RPS14 | 0.87 | 9.51E-09 | 40S ribosomal protein S14;RPS14;ortholog | 40S RIBOSOMAL PROTEIN S14 (PTHR11759:SF29) |
| **84** | ENSG00000167283 | ATP5MG | 0.87 | 1.06E-08 | ATP synthase subunit g, mitochondrial;ATP5MG;ortholog | ATP SYNTHASE SUBUNIT G 2, MITOCHONDRIAL-RELATED (PTHR12386:SF12) |
| **85** | ENSG00000168275 | COA6 | 0.87 | 6.21E-09 | Cytochrome c oxidase assembly factor 6 homolog;COA6;ortholog | CYTOCHROME C OXIDASE ASSEMBLY FACTOR 6 HOMOLOG (PTHR46690:SF1) |
| **86** | ENSG00000171858 | RPS21 | 0.87 | 9.39E-09 | 40S ribosomal protein S21;RPS21;ortholog | 40S RIBOSOMAL PROTEIN S21 (PTHR10442:SF15) |
| **87** | ENSG00000179144 | hIAN7 | 0.87 | 9.25E-09 | GTPase IMAP family member 7;GIMAP7;ortholog | GTPASE IMAP FAMILY MEMBER 7 (PTHR10903:SF163) |
| **88** | ENSG00000180964 | TCEAL8 | 0.87 | 5.86E-09 |  |  |
| **89** | ENSG00000184983 | NDUFA6 | 0.87 | 6.95E-09 | NADH dehydrogenase [ubiquinone] 1 alpha subcomplex subunit 6;NDUFA6;ortholog | NADH DEHYDROGENASE [UBIQUINONE] 1 ALPHA SUBCOMPLEX SUBUNIT 6 (PTHR12964:SF6) |
| **90** | ENSG00000109475 | RPL34 | 0.86 | 1.20E-08 | 60S ribosomal protein L34;RPL34;ortholog | 60S RIBOSOMAL PROTEIN L34 (PTHR46595:SF6) |
| **91** | ENSG00000111237 | VPS29 | 0.86 | 1.57E-08 | Vacuolar protein sorting-associated protein 29;VPS29;ortholog | VACUOLAR PROTEIN SORTING-ASSOCIATED PROTEIN 29 (PTHR11124:SF12) |
| **92** | ENSG00000121766 | ZCCHC17 | 0.86 | 2.54E-08 | Nucleolar protein of 40 kDa;ZCCHC17;ortholog | NUCLEOLAR PROTEIN OF 40 KDA (PTHR15838:SF1) |
| **93** | ENSG00000134419 | RPS15A | 0.86 | 2.42E-08 | 40S ribosomal protein S15a;RPS15A;ortholog | 40S RIBOSOMAL PROTEIN S15A (PTHR11758:SF13) |
| **94** | ENSG00000144746 | ARL6IP5 | 0.86 | 1.47E-08 | PRA1 family protein 3;ARL6IP5;ortholog | PRA1 FAMILY PROTEIN 3 (PTHR12859:SF2) |
| **95** | ENSG00000145425 | RPS3A | 0.86 | 1.56E-08 | 40S ribosomal protein S3a;RPS3A;ortholog | 40S RIBOSOMAL PROTEIN S3A (PTHR11830:SF28) |
| **96** | ENSG00000150316 | CWC15 | 0.86 | 1.62E-08 | Spliceosome-associated protein CWC15 homolog;CWC15;ortholog | SPLICEOSOME-ASSOCIATED PROTEIN CWC15 HOMOLOG (PTHR12718:SF2) |
| **97** | ENSG00000153015 | CWC27 | 0.86 | 2.51E-08 | Spliceosome-associated protein CWC27 homolog;CWC27;ortholog | SPLICEOSOME-ASSOCIATED PROTEIN CWC27 HOMOLOG (PTHR45625:SF6) |
| **98** | ENSG00000156467 | UQCRB | 0.86 | 1.39E-08 | Cytochrome b-c1 complex subunit 7;UQCRB;ortholog | CYTOCHROME B-C1 COMPLEX SUBUNIT 7 (PTHR12022:SF12) |
| **99** | ENSG00000156482 | RPL30 | 0.86 | 1.62E-08 | 60S ribosomal protein L30;RPL30;ortholog | 60S RIBOSOMAL PROTEIN L30 (PTHR11449:SF1) |
| **100** | ENSG00000162961 | DPY30 | 0.86 | 1.30E-08 |  |  |
| **101** | ENSG00000168653 | NDUFS5 | 0.86 | 1.64E-08 | NADH dehydrogenase [ubiquinone] iron-sulfur protein 5;NDUFS5;ortholog | NADH DEHYDROGENASE [UBIQUINONE] IRON-SULFUR PROTEIN 5 (PTHR15224:SF1) |
| **102** | ENSG00000169020 | ATP5ME | 0.86 | 2.53E-08 | ATP synthase subunit e, mitochondrial;ATP5ME;ortholog | ATP SYNTHASE SUBUNIT E, MITOCHONDRIAL (PTHR12427:SF1) |
| **103** | ENSG00000196683 | TOMM7 | 0.86 | 1.77E-08 | Mitochondrial import receptor subunit TOM7 homolog;TOMM7;ortholog | MITOCHONDRIAL IMPORT RECEPTOR SUBUNIT TOM7 HOMOLOG (PTHR46722:SF1) |
| **104** | ENSG00000213741 | RPS29 | 0.86 | 1.60E-08 | 40S ribosomal protein S29;RPS29;ortholog | 40S RIBOSOMAL PROTEIN S29 (PTHR12010:SF2) |
| **105** | ENSG00000230989 | HSBP1 | 0.86 | 2.01E-08 | Heat shock factor-binding protein 1;HSBP1;ortholog | HEAT SHOCK FACTOR-BINDING PROTEIN 1 (PTHR19424:SF3) |
| **106** | ENSG00000232388 | SMIM26 | 0.86 | 1.61E-08 | Small integral membrane protein 26;SMIM26;ortholog | SMALL INTEGRAL MEMBRANE PROTEIN 26 (PTHR40386:SF1) |
| **107** | ENSG00000008324 | SS18L2 | 0.85 | 5.36E-08 |  |  |
| **108** | ENSG00000008988 | RPS20 | 0.85 | 4.15E-08 | 40S ribosomal protein S20;RPS20;ortholog | 40S RIBOSOMAL PROTEIN S20 (PTHR11700:SF37) |
| **109** | ENSG00000065518 | NDUFB4 | 0.85 | 3.94E-08 | NADH dehydrogenase [ubiquinone] 1 beta subcomplex subunit 4;NDUFB4;ortholog | NADH DEHYDROGENASE [UBIQUINONE] 1 BETA SUBCOMPLEX SUBUNIT 4 (PTHR15469:SF1) |
| **110** | ENSG00000113068 | PFDN1 | 0.85 | 3.00E-08 | Prefoldin subunit 1;PFDN1;ortholog | PREFOLDIN SUBUNIT 1 (PTHR20903:SF1) |
| **111** | ENSG00000113387 | SUB1 | 0.85 | 3.56E-08 | Activated RNA polymerase II transcriptional coactivator p15;SUB1;ortholog | ACTIVATED RNA POLYMERASE II TRANSCRIPTIONAL COACTIVATOR P15 (PTHR13215:SF0) |
| **112** | ENSG00000119013 | NDUFB3 | 0.85 | 5.44E-08 | NADH dehydrogenase [ubiquinone] 1 beta subcomplex subunit 3;NDUFB3;ortholog | NADH DEHYDROGENASE [UBIQUINONE] 1 BETA SUBCOMPLEX SUBUNIT 3 (PTHR15082:SF2) |
| **113** | ENSG00000122034 | GTF3A | 0.85 | 2.79E-08 | Transcription factor IIIA;GTF3A;ortholog | TRANSCRIPTION FACTOR IIIA (PTHR46179:SF1) |
| **114** | ENSG00000135046 | ANXA1 | 0.85 | 4.67E-08 | Annexin A1;ANXA1;ortholog | ANNEXIN A1 (PTHR10502:SF17) |
| **115** | ENSG00000138326 | RPS24 | 0.85 | 3.62E-08 | 40S ribosomal protein S24;RPS24;ortholog | 40S RIBOSOMAL PROTEIN S24 (PTHR10496:SF7) |
| **116** | ENSG00000142937 | RPS8 | 0.85 | 5.38E-08 | 40S ribosomal protein S8;RPS8;ortholog | 40S RIBOSOMAL PROTEIN S8 (PTHR10394:SF12) |
| **117** | ENSG00000143575 | HAX1 | 0.85 | 2.85E-08 | HCLS1-associated protein X-1;HAX1;ortholog | HCLS1-ASSOCIATED PROTEIN X-1 (PTHR14938:SF2) |
| **118** | ENSG00000147669 | POLR2K | 0.85 | 3.96E-08 | DNA-directed RNA polymerases I, II, and III subunit RPABC4;POLR2K;ortholog | DNA-DIRECTED RNA POLYMERASES I, II, AND III SUBUNIT RPABC4 (PTHR12056:SF2) |
| **119** | ENSG00000166441 | RPL27A | 0.85 | 3.25E-08 | 60S ribosomal protein L27a;RPL27A;ortholog | 60S RIBOSOMAL PROTEIN L27A (PTHR11721:SF27) |
| **120** | ENSG00000170142 | UBE2E1 | 0.85 | 5.00E-08 | Ubiquitin-conjugating enzyme E2 E1;UBE2E1;ortholog | UBIQUITIN-CONJUGATING ENZYME E2 E1 (PTHR24068:SF70) |
| **121** | ENSG00000172586 | CHCHD1 | 0.85 | 4.90E-08 | Coiled-coil-helix-coiled-coil-helix domain-containing protein 1;CHCHD1;ortholog | COILED-COIL-HELIX-COILED-COIL-HELIX DOMAIN-CONTAINING PROTEIN 1 (PTHR31278:SF2) |
| **122** | ENSG00000227063 | RPL41P1 | 0.85 | 4.94E-08 |  |  |
| **123** | ENSG00000014641 | HEL-S-32 | 0.84 | 1.07E-07 | Malate dehydrogenase, cytoplasmic;MDH1;ortholog | MALATE DEHYDROGENASE, CYTOPLASMIC (PTHR23382:SF3) |
| **124** | ENSG00000109686 | SH3D19 | 0.84 | 7.14E-08 | SH3 domain-containing protein 19;SH3D19;ortholog | SH3 DOMAIN-CONTAINING PROTEIN 19 (PTHR14167:SF48) |
| **125** | ENSG00000116288 | HEL-S-67p | 0.84 | 6.46E-08 | Parkinson disease protein 7;PARK7;ortholog | PARKINSON DISEASE PROTEIN 7 (PTHR48094:SF16) |
| **126** | ENSG00000117091 | CD48 | 0.84 | 5.68E-08 | CD48 antigen;CD48;ortholog | CD48 ANTIGEN (PTHR12080:SF105) |
| **127** | ENSG00000118680 | MYL12B | 0.84 | 1.07E-07 | Myosin regulatory light chain 12B;MYL12B;ortholog | MYOSIN REGULATORY LIGHT CHAIN 12B (PTHR23049:SF58) |
| **128** | ENSG00000120860 | WASHC3 | 0.84 | 1.06E-07 | WASH complex subunit 3;WASHC3;ortholog | WASH COMPLEX SUBUNIT 3 (PTHR13015:SF2) |
| **129** | ENSG00000121552 | CSTA | 0.84 | 7.02E-08 | Cystatin-A;CSTA;ortholog | CYSTATIN-A (PTHR11414:SF20) |
| **130** | ENSG00000133112 | seTCTP | 0.84 | 1.12E-07 | Translationally-controlled tumor protein;TPT1;ortholog | TPT1-LIKE PROTEIN-RELATED (PTHR11991:SF9) |
| **131** | ENSG00000137947 | GTF2B | 0.84 | 6.38E-08 | Transcription initiation factor IIB;GTF2B;ortholog | TRANSCRIPTION INITIATION FACTOR IIB (PTHR11618:SF77) |
| **132** | ENSG00000149273 | RPS3 | 0.84 | 6.01E-08 | 40S ribosomal protein S3;RPS3;ortholog | 40S RIBOSOMAL PROTEIN S3 (PTHR11760:SF36) |
| **133** | ENSG00000159210 | SNF8 | 0.84 | 8.05E-08 | Vacuolar-sorting protein SNF8;SNF8;ortholog | VACUOLAR-SORTING PROTEIN SNF8 (PTHR12806:SF1) |
| **134** | ENSG00000167862 | MRPL58 | 0.84 | 9.86E-08 | Peptidyl-tRNA hydrolase ICT1, mitochondrial;MRPL58;ortholog | PEPTIDYL-TRNA HYDROLASE ICT1, MITOCHONDRIAL (PTHR11075:SF54) |
| **135** | ENSG00000188342 | GTF2F2 | 0.84 | 7.75E-08 | General transcription factor IIF subunit 2;GTF2F2;ortholog | GENERAL TRANSCRIPTION FACTOR IIF SUBUNIT 2 (PTHR10445:SF0) |
| **136** | ENSG00000270084 | GAS5-AS1 | 0.84 | 1.06E-07 |  |  |
| **137** | ENSG00000085231 | AK6 | 0.83 | 1.87E-07 | Adenylate kinase isoenzyme 6;AK6;ortholog | ADENYLATE KINASE ISOENZYME 6 (PTHR12595:SF19) |
| **138** | ENSG00000090263 | MRPS33 | 0.83 | 1.93E-07 | 28S ribosomal protein S33, mitochondrial;MRPS33;ortholog | 28S RIBOSOMAL PROTEIN S33, MITOCHONDRIAL (PTHR13362:SF2) |
| **139** | ENSG00000100410 | PHF5A | 0.83 | 1.24E-07 | PHD finger-like domain-containing protein 5A;PHF5A;ortholog | PHD FINGER-LIKE DOMAIN-CONTAINING PROTEIN 5A (PTHR13120:SF0) |
| **140** | ENSG00000120265 | PCMT1 | 0.83 | 2.13E-07 | Protein-L-isoaspartate(D-aspartate) O-methyltransferase;PCMT1;ortholog | PROTEIN-L-ISOASPARTATE(D-ASPARTATE) O-METHYLTRANSFERASE (PTHR11579:SF7) |
| **141** | ENSG00000122406 | RPL5 | 0.83 | 1.35E-07 | 60S ribosomal protein L5;RPL5;ortholog | 60S RIBOSOMAL PROTEIN L5 (PTHR23410:SF24) |
| **142** | ENSG00000128272 | ATF4 | 0.83 | 1.27E-07 | Cyclic AMP-dependent transcription factor ATF-4;ATF4;ortholog | CYCLIC AMP-DEPENDENT TRANSCRIPTION FACTOR ATF-4 (PTHR13044:SF2) |
| **143** | ENSG00000136888 | ATP6V1G1 | 0.83 | 1.81E-07 | V-type proton ATPase subunit G 1;ATP6V1G1;ortholog | V-TYPE PROTON ATPASE SUBUNIT G 1 (PTHR12713:SF12) |
| **144** | ENSG00000142541 | RPL13A | 0.83 | 1.61E-07 | 60S ribosomal protein L13a;RPL13A;ortholog | 60S RIBOSOMAL PROTEIN L13A (PTHR11545:SF30) |
| **145** | ENSG00000143621 | ILF2 | 0.83 | 1.60E-07 | Interleukin enhancer-binding factor 2;ILF2;ortholog | INTERLEUKIN ENHANCER-BINDING FACTOR 2 (PTHR46447:SF1) |
| **146** | ENSG00000149806 | FAU | 0.83 | 1.22E-07 | Ubiquitin-like protein FUBI;FAU;ortholog | UBIQUITIN-LIKE PROTEIN FUBI (PTHR10666:SF446) |
| **147** | ENSG00000156127 | BATF | 0.83 | 1.96E-07 | Basic leucine zipper transcriptional factor ATF-like;BATF;ortholog | BASIC LEUCINE ZIPPER TRANSCRIPTIONAL FACTOR ATF-LIKE (PTHR23351:SF14) |
| **148** | ENSG00000162385 | MAGOH | 0.83 | 1.13E-07 | Protein mago nashi homolog;MAGOH;ortholog | PROTEIN MAGO NASHI HOMOLOG (PTHR12638:SF2) |
| **149** | ENSG00000163479 | SSR2 | 0.83 | 1.26E-07 | Translocon-associated protein subunit beta;SSR2;ortholog | TRANSLOCON-ASSOCIATED PROTEIN SUBUNIT BETA (PTHR12861:SF3) |
| **150** | ENSG00000167088 | SNRPD1 | 0.83 | 1.78E-07 | Small nuclear ribonucleoprotein Sm D1;SNRPD1;ortholog | SMALL NUCLEAR RIBONUCLEOPROTEIN SM D1 (PTHR23338:SF18) |
| **151** | ENSG00000172239 | PAIP1 | 0.83 | 1.18E-07 | Polyadenylate-binding protein-interacting protein 1;PAIP1;ortholog | POLYADENYLATE-BINDING PROTEIN-INTERACTING PROTEIN 1 (PTHR23254:SF15) |
| **152** | ENSG00000175061 | SNHG29 | 0.83 | 1.34E-07 |  |  |
| **153** | ENSG00000176261 | ZBTB8OS | 0.83 | 1.20E-07 | Protein archease;ZBTB8OS;ortholog | PROTEIN ARCHEASE (PTHR12682:SF12) |
| **154** | ENSG00000180817 | HEL-S-66p | 0.83 | 1.80E-07 | Inorganic pyrophosphatase;PPA1;ortholog | INORGANIC PYROPHOSPHATASE (PTHR10286:SF47) |
| **155** | ENSG00000204472 | AIF1 | 0.83 | 1.94E-07 | Allograft inflammatory factor 1;AIF1;ortholog | ALLOGRAFT INFLAMMATORY FACTOR 1 (PTHR10356:SF4) |
| **156** | ENSG00000065548 | ZC3H15 | 0.82 | 3.59E-07 | Zinc finger CCCH domain-containing protein 15;ZC3H15;ortholog | ZINC FINGER CCCH DOMAIN-CONTAINING PROTEIN 15 (PTHR12681:SF0) |
| **157** | ENSG00000100442 | FKBP3 | 0.82 | 2.74E-07 | Peptidyl-prolyl cis-trans isomerase FKBP3;FKBP3;ortholog | PEPTIDYL-PROLYL CIS-TRANS ISOMERASE FKBP3 (PTHR46493:SF1) |
| **158** | ENSG00000111229 | ARPC3 | 0.82 | 2.50E-07 | Actin-related protein 2/3 complex subunit 3;ARPC3;ortholog | ACTIN-RELATED PROTEIN 2/3 COMPLEX SUBUNIT 3 (PTHR12391:SF10) |
| **159** | ENSG00000111639 | MRPL51 | 0.82 | 3.31E-07 | 39S ribosomal protein L51, mitochondrial;MRPL51;ortholog | 39S RIBOSOMAL PROTEIN L51, MITOCHONDRIAL (PTHR13409:SF0) |
| **160** | ENSG00000115514 | TXNDC9 | 0.82 | 3.68E-07 | Thioredoxin domain-containing protein 9;TXNDC9;ortholog | THIOREDOXIN DOMAIN-CONTAINING PROTEIN 9 (PTHR21148:SF11) |
| **161** | ENSG00000115944 | COX7A2L | 0.82 | 2.62E-07 | Cytochrome c oxidase subunit 7A-related protein, mitochondrial;COX7A2L;ortholog | CYTOCHROME C OXIDASE SUBUNIT 7A-RELATED PROTEIN, MITOCHONDRIAL (PTHR10510:SF2) |
| **162** | ENSG00000131100 | ATP6V1E1 | 0.82 | 3.68E-07 | V-type proton ATPase subunit E 1;ATP6V1E1;ortholog | V-TYPE PROTON ATPASE SUBUNIT E 1 (PTHR45715:SF2) |
| **163** | ENSG00000132963 | POMP | 0.82 | 3.65E-07 | Proteasome maturation protein;POMP;ortholog | PROTEASOME MATURATION PROTEIN (PTHR12828:SF3) |
| **164** | ENSG00000134333 | LDHA | 0.82 | 2.34E-07 | L-lactate dehydrogenase A chain;LDHA;ortholog | L-LACTATE DEHYDROGENASE A CHAIN (PTHR43128:SF10) |
| **165** | ENSG00000137876 | RSL24D1 | 0.82 | 2.58E-07 | Probable ribosome biogenesis protein RLP24;RSL24D1;ortholog | RIBOSOME BIOGENESIS PROTEIN RLP24-RELATED (PTHR10792:SF26) |
| **166** | ENSG00000142534 | RPS11 | 0.82 | 3.97E-07 | 40S ribosomal protein S11;RPS11;ortholog | 40S RIBOSOMAL PROTEIN S11 (PTHR10744:SF9) |
| **167** | ENSG00000143546 | S100A8 | 0.82 | 2.23E-07 | Protein S100-A8;S100A8;ortholog | PROTEIN S100-A8 (PTHR11639:SF5) |
| **168** | ENSG00000146282 | RARS2 | 0.82 | 2.43E-07 | Probable arginine--tRNA ligase, mitochondrial;RARS2;ortholog | ARGININE--TRNA LIGASE, MITOCHONDRIAL-RELATED (PTHR11956:SF11) |
| **169** | ENSG00000155438 | NIFK | 0.82 | 2.81E-07 | MKI67 FHA domain-interacting nucleolar phosphoprotein;NIFK;ortholog | MKI67 FHA DOMAIN-INTERACTING NUCLEOLAR PHOSPHOPROTEIN (PTHR46754:SF12) |
| **170** | ENSG00000166797 | CIAO2A | 0.82 | 2.73E-07 | Cytosolic iron-sulfur assembly component 2A;CIAO2A;ortholog | CYTOSOLIC IRON-SULFUR ASSEMBLY COMPONENT 2A (PTHR12377:SF6) |
| **171** | ENSG00000169019 | COMMD8 | 0.82 | 3.19E-07 | COMM domain-containing protein 8;COMMD8;ortholog | COMM DOMAIN-CONTAINING PROTEIN 8 (PTHR16231:SF0) |
| **172** | ENSG00000170860 | LSM3 | 0.82 | 3.62E-07 | U6 snRNA-associated Sm-like protein LSm3;LSM3;ortholog | U6 SNRNA-ASSOCIATED SM-LIKE PROTEIN LSM3 (PTHR13110:SF15) |
| **173** | ENSG00000173409 | ARV1 | 0.82 | 3.69E-07 | Protein ARV1;ARV1;ortholog | PROTEIN ARV1 (PTHR14467:SF0) |
| **174** | ENSG00000175324 | LSM1 | 0.82 | 2.61E-07 | U6 snRNA-associated Sm-like protein LSm1;LSM1;ortholog | U6 SNRNA-ASSOCIATED SM-LIKE PROTEIN LSM1 (PTHR15588:SF8) |
| **175** | ENSG00000181350 | LRRC75A | 0.82 | 2.27E-07 | Leucine-rich repeat-containing protein 75A;LRRC75A;ortholog | LEUCINE-RICH REPEAT-CONTAINING PROTEIN 75A (PTHR14224:SF42) |
| **176** | ENSG00000185721 | DRG1 | 0.82 | 2.84E-07 | Developmentally-regulated GTP-binding protein 1;DRG1;ortholog | DEVELOPMENTALLY-REGULATED GTP-BINDING PROTEIN 1 (PTHR43127:SF1) |
| **177** | ENSG00000067334 | DNTTIP2 | 0.81 | 4.15E-07 | Deoxynucleotidyltransferase terminal-interacting protein 2;DNTTIP2;ortholog | DEOXYNUCLEOTIDYLTRANSFERASE TERMINAL-INTERACTING PROTEIN 2 (PTHR21686:SF12) |
| **178** | ENSG00000109536 | FRG1 | 0.81 | 5.01E-07 | Protein FRG1;FRG1;ortholog | PROTEIN FRG1 (PTHR12928:SF3) |
| **179** | ENSG00000115233 | PSMD14 | 0.81 | 4.60E-07 | 26S proteasome non-ATPase regulatory subunit 14;PSMD14;ortholog | 26S PROTEASOME NON-ATPASE REGULATORY SUBUNIT 14 (PTHR10410:SF5) |
| **180** | ENSG00000117543 | DPH5 | 0.81 | 4.80E-07 | Diphthine methyl ester synthase;DPH5;ortholog | DIPHTHINE METHYL ESTER SYNTHASE (PTHR10882:SF0) |
| **181** | ENSG00000131143 | COX4I1 | 0.81 | 5.93E-07 | Cytochrome c oxidase subunit 4 isoform 1, mitochondrial;COX4I1;ortholog | CYTOCHROME C OXIDASE SUBUNIT 4 ISOFORM 1, MITOCHONDRIAL (PTHR10707:SF12) |
| **182** | ENSG00000144713 | RPL32 | 0.81 | 6.10E-07 | 60S ribosomal protein L32;RPL32;ortholog | 60S RIBOSOMAL PROTEIN L32 (PTHR23413:SF6) |
| **183** | ENSG00000145088 | EAF2 | 0.81 | 4.13E-07 | ELL-associated factor 2;EAF2;ortholog | ELL-ASSOCIATED FACTOR 2 (PTHR15970:SF7) |
| **184** | ENSG00000161970 | RPL26 | 0.81 | 4.77E-07 | 60S ribosomal protein L26;RPL26;ortholog | 60S RIBOSOMAL PROTEIN L26 (PTHR11143:SF11) |
| **185** | ENSG00000164404 | GDF9 | 0.81 | 5.34E-07 | Growth/differentiation factor 9;GDF9;ortholog | GROWTH/DIFFERENTIATION FACTOR 9 (PTHR11848:SF19) |
| **186** | ENSG00000166710 | B2M | 0.81 | 6.25E-07 | Beta-2-microglobulin;B2M;ortholog | BETA-2-MICROGLOBULIN (PTHR19944:SF62) |
| **187** | ENSG00000168028 | RPSA | 0.81 | 5.58E-07 | 40S ribosomal protein SA;RPSA;ortholog | 40S RIBOSOMAL PROTEIN SA (PTHR11489:SF17) |
| **188** | ENSG00000169740 | ZNF32 | 0.81 | 4.11E-07 | Zinc finger protein 32;ZNF32;ortholog | ZINC FINGER PROTEIN 32 (PTHR23226:SF214) |
| **189** | ENSG00000169976 | SF3B5 | 0.81 | 6.08E-07 | Splicing factor 3B subunit 5;SF3B5;ortholog | SPLICING FACTOR 3B SUBUNIT 5 (PTHR20978:SF0) |
| **190** | ENSG00000170310 | STX8 | 0.81 | 5.41E-07 | Syntaxin-8;STX8;ortholog | SYNTAXIN-8 (PTHR19957:SF285) |
| **191** | ENSG00000205302 | SNX2 | 0.81 | 4.96E-07 | Sorting nexin-2;SNX2;ortholog | SORTING NEXIN-2 (PTHR10555:SF31) |
| **192** | ENSG00000086061 | DNAJA1 | 0.8 | 1.21E-06 | DnaJ homolog subfamily A member 1;DNAJA1;ortholog | DNAJ HOMOLOG SUBFAMILY A MEMBER 1 (PTHR43888:SF8) |
| **193** | ENSG00000099795 | NDUFB7 | 0.8 | 9.14E-07 | NADH dehydrogenase [ubiquinone] 1 beta subcomplex subunit 7;NDUFB7;ortholog | NADH DEHYDROGENASE [UBIQUINONE] 1 BETA SUBCOMPLEX SUBUNIT 7 (PTHR20900:SF0) |
| **194** | ENSG00000103671 | TRIP4 | 0.8 | 1.07E-06 | Activating signal cointegrator 1;TRIP4;ortholog | ACTIVATING SIGNAL COINTEGRATOR 1 (PTHR12963:SF4) |
| **195** | ENSG00000105254 | TBCB | 0.8 | 1.18E-06 | Tubulin-folding cofactor B;TBCB;ortholog | TUBULIN-FOLDING COFACTOR B (PTHR18916:SF85) |
| **196** | ENSG00000118181 | RPS25 | 0.8 | 9.34E-07 | 40S ribosomal protein S25;RPS25;ortholog | 40S RIBOSOMAL PROTEIN S25 (PTHR12850:SF18) |
| **197** | ENSG00000122873 | CISD1 | 0.8 | 1.03E-06 | CDGSH iron-sulfur domain-containing protein 1;CISD1;ortholog | CDGSH IRON-SULFUR DOMAIN-CONTAINING PROTEIN 1 (PTHR13680:SF42) |
| **198** | ENSG00000125351 | UPF3B | 0.8 | 1.10E-06 | Regulator of nonsense transcripts 3B;UPF3B;ortholog | REGULATOR OF NONSENSE TRANSCRIPTS 3B (PTHR13112:SF1) |
| **199** | ENSG00000126698 | DNAJC8 | 0.8 | 1.11E-06 | DnaJ homolog subfamily C member 8;DNAJC8;ortholog | DNAJ HOMOLOG SUBFAMILY C MEMBER 8 (PTHR15606:SF7) |
| **200** | ENSG00000137547 | MRPL15 | 0.8 | 7.64E-07 | 39S ribosomal protein L15, mitochondrial;MRPL15;ortholog | 39S RIBOSOMAL PROTEIN L15, MITOCHONDRIAL (PTHR12934:SF11) |
| **201** | ENSG00000141428 | C18orf21 | 0.8 | 1.12E-06 | UPF0711 protein C18orf21;C18orf21;ortholog | UPF0711 PROTEIN C18ORF21 (PTHR31402:SF2) |
| **202** | ENSG00000142507 | PSMB6 | 0.8 | 7.74E-07 | Proteasome subunit beta type-6;PSMB6;ortholog | PROTEASOME SUBUNIT BETA TYPE-6 (PTHR11599:SF46) |
| **203** | ENSG00000154582 | ELOC | 0.8 | 1.04E-06 | Elongin-C;ELOC;ortholog | ELONGIN-C (PTHR20648:SF11) |
| **204** | ENSG00000166226 | HEL-S-100n | 0.8 | 7.48E-07 | T-complex protein 1 subunit beta;CCT2;ortholog | T-COMPLEX PROTEIN 1 SUBUNIT BETA (PTHR11353:SF23) |
| **205** | ENSG00000167863 | ATP5PD | 0.8 | 9.73E-07 | ATP synthase subunit d, mitochondrial;ATP5PD;ortholog | ATP SYNTHASE SUBUNIT D, MITOCHONDRIAL (PTHR12700:SF17) |
| **206** | ENSG00000174173 | TRMT10C | 0.8 | 1.12E-06 | tRNA methyltransferase 10 homolog C;TRMT10C;ortholog | TRNA METHYLTRANSFERASE 10 HOMOLOG C (PTHR13563:SF5) |
| **207** | ENSG00000101132 | PFDN4 | 0.79 | 1.77E-06 | Prefoldin subunit 4;PFDN4;ortholog | PREFOLDIN SUBUNIT 4 (PTHR21100:SF9) |
| **208** | ENSG00000104408 | EIF3E | 0.79 | 1.55E-06 | Eukaryotic translation initiation factor 3 subunit E;EIF3E;ortholog | EUKARYOTIC TRANSLATION INITIATION FACTOR 3 SUBUNIT E (PTHR10317:SF3) |
| **209** | ENSG00000104529 | EEF1D | 0.79 | 1.30E-06 | Elongation factor 1-delta;EEF1D;ortholog | ELONGATION FACTOR 1-DELTA (PTHR11595:SF26) |
| **210** | ENSG00000105819 | PMPCB | 0.79 | 2.00E-06 | Mitochondrial-processing peptidase subunit beta;PMPCB;ortholog | MITOCHONDRIAL-PROCESSING PEPTIDASE SUBUNIT BETA (PTHR11851:SF103) |
| **211** | ENSG00000106153 | CHCHD2 | 0.79 | 1.32E-06 | Coiled-coil-helix-coiled-coil-helix domain-containing protein 2;CHCHD2;ortholog | COILED-COIL-HELIX-COILED-COIL-HELIX DOMAIN-CONTAINING PROTEIN 2-RELATED (PTHR13523:SF3) |
| **212** | ENSG00000110944 | IL23A | 0.79 | 1.88E-06 | Interleukin-23 subunit alpha;IL23A;ortholog | INTERLEUKIN-23 SUBUNIT ALPHA (PTHR15947:SF0) |
| **213** | ENSG00000115350 | POLE4 | 0.79 | 1.33E-06 | DNA polymerase epsilon subunit 4;POLE4;ortholog | DNA POLYMERASE EPSILON SUBUNIT 4 (PTHR10252:SF79) |
| **214** | ENSG00000129824 | RPS4Y1 | 0.79 | 1.80E-06 | 40S ribosomal protein S4, Y isoform 1;RPS4Y1;ortholog | 40S RIBOSOMAL PROTEIN S4, Y ISOFORM 1 (PTHR11581:SF8) |
| **215** | ENSG00000131475 | VPS25 | 0.79 | 1.46E-06 | Vacuolar protein-sorting-associated protein 25;VPS25;ortholog | VACUOLAR PROTEIN-SORTING-ASSOCIATED PROTEIN 25 (PTHR13149:SF0) |
| **216** | ENSG00000136143 | SUCLA2 | 0.79 | 1.27E-06 | Succinate--CoA ligase [ADP-forming] subunit beta, mitochondrial;SUCLA2;ortholog | SUCCINATE--COA LIGASE [ADP-FORMING] SUBUNIT BETA, MITOCHONDRIAL (PTHR11815:SF14) |
| **217** | ENSG00000136522 | MRPL47 | 0.79 | 1.25E-06 | 39S ribosomal protein L47, mitochondrial;MRPL47;ortholog | 39S RIBOSOMAL PROTEIN L47, MITOCHONDRIAL (PTHR21183:SF18) |
| **218** | ENSG00000140307 | GTF2A2 | 0.79 | 2.00E-06 | Transcription initiation factor IIA subunit 2;GTF2A2;ortholog | TRANSCRIPTION INITIATION FACTOR IIA SUBUNIT 2 (PTHR10966:SF5) |
| **219** | ENSG00000140379 | BCL2A1 | 0.79 | 1.31E-06 | Bcl-2-related protein A1;BCL2A1;ortholog | BCL-2-RELATED PROTEIN A1 (PTHR11256:SF10) |
| **220** | ENSG00000143222 | UFC1 | 0.79 | 1.32E-06 | Ubiquitin-fold modifier-conjugating enzyme 1;UFC1;ortholog | UBIQUITIN-FOLD MODIFIER-CONJUGATING ENZYME 1 (PTHR12921:SF0) |
| **221** | ENSG00000143256 | PFDN2 | 0.79 | 1.23E-06 | Prefoldin subunit 2;PFDN2;ortholog | PREFOLDIN SUBUNIT 2 (PTHR13303:SF0) |
| **222** | ENSG00000154719 | MRPL39 | 0.79 | 1.85E-06 | 39S ribosomal protein L39, mitochondrial;MRPL39;ortholog | 39S RIBOSOMAL PROTEIN L39, MITOCHONDRIAL (PTHR42753:SF9) |
| **223** | ENSG00000156508 | EEF1A1 | 0.79 | 2.02E-06 | Elongation factor 1-alpha 1;EEF1A1;ortholog | ELONGATION FACTOR 1-ALPHA 1-RELATED (PTHR23115:SF222) |
| **224** | ENSG00000159377 | PSMB4 | 0.79 | 1.91E-06 | Proteasome subunit beta type-4;PSMB4;ortholog | PROTEASOME SUBUNIT BETA TYPE-4 (PTHR11599:SF5) |
| **225** | ENSG00000160799 | CCDC12 | 0.79 | 1.89E-06 | Coiled-coil domain-containing protein 12;CCDC12;ortholog | COILED-COIL DOMAIN-CONTAINING PROTEIN 12 (PTHR31551:SF1) |
| **226** | ENSG00000165502 | RPL36AL | 0.79 | 1.56E-06 | 60S ribosomal protein L36a-like;RPL36AL;ortholog | 60S RIBOSOMAL PROTEIN L36A-LIKE (PTHR10369:SF45) |
| **227** | ENSG00000175581 | MRPL48 | 0.79 | 1.30E-06 | 39S ribosomal protein L48, mitochondrial;MRPL48;ortholog | 39S RIBOSOMAL PROTEIN L48, MITOCHONDRIAL (PTHR13473:SF0) |
| **228** | ENSG00000182004 | SNRPE | 0.79 | 1.26E-06 | Small nuclear ribonucleoprotein E;SNRPE;ortholog | SMALL NUCLEAR RIBONUCLEOPROTEIN E (PTHR11193:SF9) |
| **229** | ENSG00000185608 | MRPL40 | 0.79 | 1.32E-06 | 39S ribosomal protein L40, mitochondrial;MRPL40;ortholog | 39S RIBOSOMAL PROTEIN L40, MITOCHONDRIAL (PTHR13359:SF2) |
| **230** | ENSG00000188243 | COMMD6 | 0.79 | 1.76E-06 | COMM domain-containing protein 6;COMMD6;ortholog | COMM DOMAIN-CONTAINING PROTEIN 6 (PTHR16231:SF5) |
| **231** | ENSG00000215375 | MYL5 | 0.79 | 1.30E-06 | Myosin light chain 5;MYL5;ortholog | MYOSIN LIGHT CHAIN 5 (PTHR23049:SF8) |
| **232** | ENSG00000089289 | IGBP1 | 0.78 | 2.82E-06 | Immunoglobulin-binding protein 1;IGBP1;ortholog | IMMUNOGLOBULIN-BINDING PROTEIN 1 (PTHR10933:SF12) |
| **233** | ENSG00000110852 | CLEC2B | 0.78 | 2.48E-06 | C-type lectin domain family 2 member B;CLEC2B;ortholog | C-TYPE LECTIN DOMAIN FAMILY 2 MEMBER B (PTHR45710:SF15) |
| **234** | ENSG00000111142 | METAP2 | 0.78 | 2.54E-06 | Methionine aminopeptidase 2;METAP2;ortholog | METHIONINE AMINOPEPTIDASE 2 (PTHR45777:SF2) |
| **235** | ENSG00000113643 | RARS1 | 0.78 | 2.58E-06 | Arginine--tRNA ligase, cytoplasmic;RARS1;ortholog | ARGININE--TRNA LIGASE, CYTOPLASMIC (PTHR11956:SF5) |
| **236** | ENSG00000114125 | RNF7 | 0.78 | 2.67E-06 | RING-box protein 2;RNF7;ortholog | E3 UBIQUITIN-PROTEIN LIGASE RBX1-RELATED (PTHR11210:SF2) |
| **237** | ENSG00000114686 | MRPL3 | 0.78 | 2.97E-06 | 39S ribosomal protein L3, mitochondrial;MRPL3;ortholog | 39S RIBOSOMAL PROTEIN L3, MITOCHONDRIAL (PTHR11229:SF13) |
| **238** | ENSG00000132646 | PCNA | 0.78 | 2.38E-06 | Proliferating cell nuclear antigen;PCNA;ortholog | PROLIFERATING CELL NUCLEAR ANTIGEN (PTHR11352:SF5) |
| **239** | ENSG00000135211 | TMEM60 | 0.78 | 2.11E-06 | Transmembrane protein 60;TMEM60;ortholog | TRANSMEMBRANE PROTEIN 60 (PTHR13568:SF4) |
| **240** | ENSG00000138385 | SSB | 0.78 | 2.26E-06 | Lupus La protein;SSB;ortholog | LUPUS LA PROTEIN (PTHR22792:SF158) |
| **241** | ENSG00000149196 | HIKESHI | 0.78 | 2.62E-06 | Protein Hikeshi;HIKESHI;ortholog | PROTEIN HIKESHI (PTHR12925:SF0) |
| **242** | ENSG00000151500 | THYN1 | 0.78 | 2.12E-06 | Thymocyte nuclear protein 1;THYN1;ortholog | THYMOCYTE NUCLEAR PROTEIN 1 (PTHR14087:SF7) |
| **243** | ENSG00000153140 | CETN3 | 0.78 | 2.60E-06 | Centrin-3;CETN3;ortholog | CENTRIN-3 (PTHR23050:SF325) |
| **244** | ENSG00000163541 | SUCLG1 | 0.78 | 2.49E-06 | Succinate--CoA ligase [ADP/GDP-forming] subunit alpha, mitochondrial;SUCLG1;ortholog | SUCCINATE--COA LIGASE [ADP/GDP-FORMING] SUBUNIT ALPHA, MITOCHONDRIAL (PTHR11117:SF2) |
| **245** | ENSG00000166595 | CIAO2B | 0.78 | 2.78E-06 | Cytosolic iron-sulfur assembly component 2B;CIAO2B;ortholog | CYTOSOLIC IRON-SULFUR ASSEMBLY COMPONENT 2B (PTHR12377:SF0) |
| **246** | ENSG00000168259 | DNAJC7 | 0.78 | 2.39E-06 | DnaJ homolog subfamily C member 7;DNAJC7;ortholog | DNAJ HOMOLOG SUBFAMILY C MEMBER 7 (PTHR45188:SF2) |
| **247** | ENSG00000177889 | HEL-S-71 | 0.78 | 2.31E-06 | Ubiquitin-conjugating enzyme E2 N;UBE2N;ortholog | UBIQUITIN-CONJUGATING ENZYME E2 N (PTHR24068:SF152) |
| **248** | ENSG00000186132 | C2orf76 | 0.78 | 2.70E-06 | UPF0538 protein C2orf76;C2orf76;ortholog | UPF0538 PROTEIN C2ORF76 (PTHR18444:SF9) |
| **249** | ENSG00000186714 | CCDC73 | 0.78 | 2.22E-06 | Coiled-coil domain-containing protein 73;CCDC73;ortholog | COILED-COIL DOMAIN-CONTAINING PROTEIN 73 (PTHR28660:SF1) |
| **250** | ENSG00000231663 | COA6-AS1 | 0.78 | 2.31E-06 |  |  |
| **251** | ENSG00000234741 | GAS5 | 0.78 | 2.68E-06 |  |  |
| **252** | ENSG00000241468 | ATP5MF | 0.78 | 2.10E-06 | ATP synthase subunit f, mitochondrial;ATP5MF;ortholog | ATP SYNTHASE SUBUNIT F, MITOCHONDRIAL (PTHR13080:SF18) |
| **253** | ENSG00000241837 | ATP5PO | 0.78 | 2.22E-06 | ATP synthase subunit O, mitochondrial;ATP5PO;ortholog | ATP SYNTHASE SUBUNIT O, MITOCHONDRIAL (PTHR11910:SF21) |
| **254** | ENSG00000254999 | BRK1 | 0.78 | 2.34E-06 | Protein BRICK1;BRK1;ortholog | PROTEIN BRICK1 (PTHR33668:SF1) |
| **255** | ENSG00000072849 | DERL2 | 0.77 | 3.66E-06 | Derlin-2;DERL2;ortholog | DERLIN-2 (PTHR11009:SF5) |
| **256** | ENSG00000114209 | PDCD10 | 0.77 | 3.67E-06 | Programmed cell death protein 10;PDCD10;ortholog | PROGRAMMED CELL DEATH PROTEIN 10 (PTHR13250:SF1) |
| **257** | ENSG00000126768 | TIMM17B | 0.77 | 3.58E-06 | Mitochondrial import inner membrane translocase subunit Tim17-B;TIMM17B;ortholog | MITOCHONDRIAL IMPORT INNER MEMBRANE TRANSLOCASE SUBUNIT TIM17-B (PTHR10485:SF2) |
| **258** | ENSG00000128609 | NDUFA5 | 0.77 | 3.42E-06 | NADH dehydrogenase [ubiquinone] 1 alpha subcomplex subunit 5;NDUFA5;ortholog | NADH DEHYDROGENASE [UBIQUINONE] 1 ALPHA SUBCOMPLEX SUBUNIT 5 (PTHR12653:SF0) |
| **259** | ENSG00000170584 | NUDCD2 | 0.77 | 3.54E-06 | NudC domain-containing protein 2;NUDCD2;ortholog | NUDC DOMAIN-CONTAINING PROTEIN 2 (PTHR12356:SF18) |
| **260** | ENSG00000242485 | MRPL20 | 0.77 | 3.81E-06 | 39S ribosomal protein L20, mitochondrial;MRPL20;ortholog | 39S RIBOSOMAL PROTEIN L20, MITOCHONDRIAL (PTHR10986:SF16) |
| **261** | ENSG00000055609 | KMT2C | -0.77 | 3.77E-06 | Histone-lysine N-methyltransferase 2C;KMT2C;ortholog | HISTONE-LYSINE N-METHYLTRANSFERASE 2C (PTHR45888:SF1) |
| **262** | ENSG00000169057 | MECP2 | -0.77 | 3.66E-06 | Methyl-CpG-binding protein 2;MECP2;ortholog | METHYL-CPG-BINDING PROTEIN 2 (PTHR15074:SF6) |
| **263** | ENSG00000101109 | STK4 | -0.78 | 2.86E-06 | Serine/threonine-protein kinase 4;STK4;ortholog | SERINE/THREONINE-PROTEIN KINASE 4 (PTHR48015:SF32) |
| **264** | ENSG00000110713 | NUP98 | -0.78 | 3.16E-06 | Nuclear pore complex protein Nup98-Nup96;NUP98;ortholog | NUCLEAR PORE COMPLEX PROTEIN NUP98-NUP96 (PTHR23198:SF6) |
| **265** | ENSG00000115935 | WIPF1 | -0.78 | 3.34E-06 | WAS/WASL-interacting protein family member 1;WIPF1;ortholog | WAS/WASL-INTERACTING PROTEIN FAMILY MEMBER 1 (PTHR48226:SF1) |
| **266** | ENSG00000119138 | KLF9 | -0.78 | 2.73E-06 | Krueppel-like factor 9;KLF9;ortholog | KRUEPPEL-LIKE FACTOR 9 (PTHR23235:SF132) |
| **267** | ENSG00000120690 | ELF1 | -0.78 | 2.23E-06 | ETS-related transcription factor Elf-1;ELF1;ortholog | ETS-RELATED TRANSCRIPTION FACTOR ELF-1 (PTHR11849:SF156) |
| **268** | ENSG00000153561 | RMND5A | -0.78 | 2.07E-06 | E3 ubiquitin-protein transferase RMND5A;RMND5A;ortholog | E3 UBIQUITIN-PROTEIN TRANSFERASE RMND5A (PTHR12170:SF5) |
| **269** | ENSG00000156675 | RAB11FIP1 | -0.78 | 2.82E-06 | Rab11 family-interacting protein 1;RAB11FIP1;ortholog | RAB11 FAMILY-INTERACTING PROTEIN 1 (PTHR15746:SF22) |
| **270** | ENSG00000163349 | HIPK1 | -0.78 | 2.90E-06 | Homeodomain-interacting protein kinase 1;HIPK1;ortholog | HOMEODOMAIN-INTERACTING PROTEIN KINASE 1 (PTHR24058:SF43) |
| **271** | ENSG00000169375 | SIN3A | -0.78 | 2.21E-06 | Paired amphipathic helix protein Sin3a;SIN3A;ortholog | PAIRED AMPHIPATHIC HELIX PROTEIN SIN3A (PTHR12346:SF2) |
| **272** | ENSG00000174579 | MSL2 | -0.78 | 2.82E-06 | E3 ubiquitin-protein ligase MSL2;MSL2;ortholog | E3 UBIQUITIN-PROTEIN LIGASE MSL2 (PTHR16048:SF3) |
| **273** | ENSG00000181826 | RELL1 | -0.78 | 2.76E-06 | RELT-like protein 1;RELL1;ortholog | RELT-LIKE PROTEIN 1 (PTHR31037:SF1) |
| **274** | ENSG00000198265 | HELZ | -0.78 | 3.20E-06 | Probable helicase with zinc finger domain;HELZ;ortholog | HELICASE WITH ZINC FINGER DOMAIN-RELATED (PTHR10887:SF365) |
| **275** | ENSG00000198646 | NCOA6 | -0.78 | 3.13E-06 | Nuclear receptor coactivator 6;NCOA6;ortholog | NUCLEAR RECEPTOR COACTIVATOR 6 (PTHR15690:SF0) |
| **276** | ENSG00000198742 | SMURF1 | -0.78 | 2.51E-06 | E3 ubiquitin-protein ligase SMURF1;SMURF1;ortholog | E3 UBIQUITIN-PROTEIN LIGASE SMURF1 (PTHR11254:SF293) |
| **277** | ENSG00000048828 | FAM120A | -0.79 | 1.26E-06 | Constitutive coactivator of PPAR-gamma-like protein 1;FAM120A;ortholog | CONSTITUTIVE COACTIVATOR OF PPAR-GAMMA-LIKE PROTEIN 1 (PTHR15976:SF14) |
| **278** | ENSG00000100207 | TCF20 | -0.79 | 1.62E-06 | Transcription factor 20;TCF20;ortholog | TRANSCRIPTION FACTOR 20 (PTHR14955:SF7) |
| **279** | ENSG00000100697 | DICER1 | -0.79 | 1.41E-06 | Endoribonuclease Dicer;DICER1;ortholog | ENDORIBONUCLEASE DICER (PTHR14950:SF37) |
| **280** | ENSG00000103489 | XYLT1 | -0.79 | 1.65E-06 | Xylosyltransferase 1;XYLT1;ortholog | XYLOSYLTRANSFERASE 1 (PTHR46025:SF2) |
| **281** | ENSG00000107929 | LARP4B | -0.79 | 1.24E-06 | La-related protein 4B;LARP4B;ortholog | LA-RELATED PROTEIN 4B (PTHR22792:SF43) |
| **282** | ENSG00000151702 | FLI1 | -0.79 | 1.68E-06 | Friend leukemia integration 1 transcription factor;FLI1;ortholog | FRIEND LEUKEMIA INTEGRATION 1 TRANSCRIPTION FACTOR (PTHR11849:SF161) |
| **283** | ENSG00000167548 | KMT2D | -0.79 | 1.32E-06 | Histone-lysine N-methyltransferase 2D;KMT2D;ortholog | HISTONE-LYSINE N-METHYLTRANSFERASE 2D (PTHR45888:SF2) |
| **284** | ENSG00000185950 | IRS2 | -0.79 | 1.79E-06 | Insulin receptor substrate 2;IRS2;ortholog | INSULIN RECEPTOR SUBSTRATE 2 (PTHR10614:SF7) |
| **285** | ENSG00000189079 | ARID2 | -0.79 | 1.94E-06 | AT-rich interactive domain-containing protein 2;ARID2;ortholog | AT-RICH INTERACTIVE DOMAIN-CONTAINING PROTEIN 2 (PTHR46691:SF1) |
| **286** | ENSG00000234617 | SNRK-AS1 | -0.79 | 1.44E-06 |  |  |
| **287** | ENSG00000247595 | SPTY2D1OS | -0.79 | 1.47E-06 |  |  |
| **288** | ENSG00000063601 | MTMR1 | -0.8 | 1.05E-06 | Myotubularin-related protein 1;MTMR1;ortholog | MYOTUBULARIN-RELATED PROTEIN 1 (PTHR10807:SF40) |
| **289** | ENSG00000074755 | ZZEF1 | -0.8 | 9.47E-07 | Zinc finger ZZ-type and EF-hand domain-containing protein 1;ZZEF1;ortholog | ZINC FINGER ZZ-TYPE AND EF-HAND DOMAIN-CONTAINING PROTEIN 1 (PTHR22772:SF4) |
| **290** | ENSG00000100354 | TNRC6B | -0.8 | 1.14E-06 | Trinucleotide repeat-containing gene 6B protein;TNRC6B;ortholog | TRINUCLEOTIDE REPEAT-CONTAINING GENE 6B PROTEIN (PTHR13020:SF32) |
| **291** | ENSG00000103657 | HERC1 | -0.8 | 7.17E-07 | Probable E3 ubiquitin-protein ligase HERC1;HERC1;ortholog | E3 UBIQUITIN-PROTEIN LIGASE HERC1-RELATED (PTHR22870:SF155) |
| **292** | ENSG00000109787 | KLF3 | -0.8 | 7.68E-07 | Krueppel-like factor 3;KLF3;ortholog | KRUEPPEL-LIKE FACTOR 3 (PTHR23235:SF48) |
| **293** | ENSG00000110395 | CBL | -0.8 | 1.21E-06 | E3 ubiquitin-protein ligase CBL;CBL;ortholog | E3 UBIQUITIN-PROTEIN LIGASE CBL (PTHR23007:SF5) |
| **294** | ENSG00000116539 | ASH1L | -0.8 | 7.34E-07 | Histone-lysine N-methyltransferase ASH1L;ASH1L;ortholog | HISTONE-LYSINE N-METHYLTRANSFERASE ASH1L (PTHR46147:SF1) |
| **295** | ENSG00000118482 | PHF3 | -0.8 | 7.85E-07 | PHD finger protein 3;PHF3;ortholog | PHD FINGER PROTEIN 3 (PTHR11477:SF10) |
| **296** | ENSG00000132549 | VPS13B | -0.8 | 1.03E-06 | Vacuolar protein sorting-associated protein 13B;VPS13B;ortholog | VACUOLAR PROTEIN SORTING-ASSOCIATED PROTEIN 13B (PTHR12517:SF0) |
| **297** | ENSG00000138802 | SEC24B | -0.8 | 9.82E-07 | Protein transport protein Sec24B;SEC24B;ortholog | PROTEIN TRANSPORT PROTEIN SEC24B (PTHR13803:SF42) |
| **298** | ENSG00000157514 | TSC22D3 | -0.8 | 8.63E-07 | TSC22 domain family protein 3;TSC22D3;ortholog | TSC22 DOMAIN FAMILY PROTEIN 3 (PTHR12348:SF24) |
| **299** | ENSG00000160179 | ABCG1 | -0.8 | 8.86E-07 | ATP-binding cassette sub-family G member 1;ABCG1;ortholog | ATP-BINDING CASSETTE SUB-FAMILY G MEMBER 1 (PTHR48041:SF90) |
| **300** | ENSG00000005339 | CREBBP | -0.81 | 6.11E-07 | CREB-binding protein;CREBBP;ortholog | CREB-BINDING PROTEIN (PTHR13808:SF34) |
| **301** | ENSG00000087589 | CASS4 | -0.81 | 5.29E-07 | Cas scaffolding protein family member 4;CASS4;ortholog | CAS SCAFFOLDING PROTEIN FAMILY MEMBER 4 (PTHR10654:SF19) |
| **302** | ENSG00000116731 | PRDM2 | -0.81 | 5.30E-07 | PR domain zinc finger protein 2;PRDM2;ortholog | PR DOMAIN ZINC FINGER PROTEIN 2 (PTHR16515:SF37) |
| **303** | ENSG00000158636 | EMSY | -0.81 | 4.10E-07 | BRCA2-interacting transcriptional repressor EMSY;EMSY;ortholog | BRCA2-INTERACTING TRANSCRIPTIONAL REPRESSOR EMSY (PTHR16500:SF3) |
| **304** | ENSG00000160218 | TRAPPC10 | -0.81 | 4.38E-07 | Trafficking protein particle complex subunit 10;TRAPPC10;ortholog | TRAFFICKING PROTEIN PARTICLE COMPLEX SUBUNIT 10 (PTHR13251:SF3) |
| **305** | ENSG00000180370 | PAK2 | -0.81 | 4.45E-07 | Serine/threonine-protein kinase PAK 2;PAK2;ortholog | SERINE/THREONINE-PROTEIN KINASE PAK 2 (PTHR48015:SF22) |
| **306** | ENSG00000008083 | JARID2 | -0.82 | 3.61E-07 | Protein Jumonji;JARID2;ortholog | PROTEIN JUMONJI (PTHR10694:SF113) |
| **307** | ENSG00000109756 | RAPGEF2 | -0.82 | 3.69E-07 | Rap guanine nucleotide exchange factor 2;RAPGEF2;ortholog | RAP GUANINE NUCLEOTIDE EXCHANGE FACTOR 2 (PTHR23113:SF217) |
| **308** | ENSG00000133639 | BTG1 | -0.82 | 2.87E-07 | Protein BTG1;BTG1;ortholog | PROTEIN BTG1 (PTHR22978:SF30) |
| **309** | ENSG00000149503 | INCENP | -0.82 | 2.46E-07 | Inner centromere protein;INCENP;ortholog | INNER CENTROMERE PROTEIN (PTHR13142:SF1) |
| **310** | ENSG00000162434 | JAK1 | -0.82 | 2.66E-07 | Tyrosine-protein kinase JAK1;JAK1;ortholog | TYROSINE-PROTEIN KINASE JAK1 (PTHR45807:SF5) |
| **311** | ENSG00000173273 | TNKS | -0.82 | 2.76E-07 | Poly [ADP-ribose] polymerase tankyrase-1;TNKS;ortholog | POLY [ADP-RIBOSE] POLYMERASE TANKYRASE-1 (PTHR24180:SF3) |
| **312** | ENSG00000229619 | MBNL1-AS1 | -0.82 | 2.35E-07 |  |  |
| **313** | ENSG00000233766 | CAVIN2-AS1 | -0.82 | 3.65E-07 |  |  |
| **314** | ENSG00000100393 | EP300 | -0.83 | 1.72E-07 | Histone acetyltransferase p300;EP300;ortholog | HISTONE ACETYLTRANSFERASE P300 (PTHR13808:SF29) |
| **315** | ENSG00000101126 | ADNP | -0.83 | 1.14E-07 | Activity-dependent neuroprotector homeobox protein;ADNP;ortholog | ACTIVITY-DEPENDENT NEUROPROTECTOR HOMEOBOX PROTEIN (PTHR15740:SF1) |
| **316** | ENSG00000124789 | NUP153 | -0.83 | 1.88E-07 | Nuclear pore complex protein Nup153;NUP153;ortholog | NUCLEAR PORE COMPLEX PROTEIN NUP153 (PTHR23193:SF23) |
| **317** | ENSG00000147133 | TAF1 | -0.83 | 1.57E-07 | Transcription initiation factor TFIID subunit 1;TAF1;ortholog | TRANSCRIPTION INITIATION FACTOR TFIID SUBUNIT 1 (PTHR13900:SF1) |
| **318** | ENSG00000155111 | CDK19 | -0.83 | 1.64E-07 | Cyclin-dependent kinase 19;CDK19;ortholog | CYCLIN-DEPENDENT KINASE 19 (PTHR24056:SF495) |
| **319** | ENSG00000165671 | NSD1 | -0.83 | 1.19E-07 | Histone-lysine N-methyltransferase, H3 lysine-36 specific;NSD1;ortholog | HISTONE-LYSINE N-METHYLTRANSFERASE, H3 LYSINE-36 SPECIFIC (PTHR22884:SF312) |
| **320** | ENSG00000169926 | KLF13 | -0.83 | 1.61E-07 | Krueppel-like factor 13;KLF13;ortholog | KRUEPPEL-LIKE FACTOR 13 (PTHR23235:SF21) |
| **321** | ENSG00000204120 | GIGYF2 | -0.83 | 1.87E-07 | GRB10-interacting GYF protein 2;GIGYF2;ortholog | GRB10-INTERACTING GYF PROTEIN 2 (PTHR14445:SF38) |
| **322** | ENSG00000224470 | ATXN1L | -0.83 | 1.39E-07 | Ataxin-1-like;ATXN1L;ortholog | ATAXIN-1-LIKE (PTHR13392:SF6) |
| **323** | ENSG00000233554 | B4GALT1-AS1 | -0.83 | 1.92E-07 |  |  |
| **324** | ENSG00000053254 | CHES1 | -0.84 | 1.03E-07 | Forkhead box protein N3;FOXN3;ortholog | FORKHEAD BOX PROTEIN N3 (PTHR13962:SF20) |
| **325** | ENSG00000083168 | KAT6A | -0.84 | 7.38E-08 | Histone acetyltransferase KAT6A;KAT6A;ortholog | HISTONE ACETYLTRANSFERASE KAT6A (PTHR10615:SF26) |
| **326** | ENSG00000139718 | SETD1B | -0.84 | 8.35E-08 | Histone-lysine N-methyltransferase SETD1B;SETD1B;ortholog | HISTONE-LYSINE N-METHYLTRANSFERASE SETD1B (PTHR45814:SF1) |
| **327** | ENSG00000157404 | KIT | -0.84 | 5.85E-08 | Mast/stem cell growth factor receptor Kit;KIT;ortholog | MAST/STEM CELL GROWTH FACTOR RECEPTOR KIT (PTHR24416:SF46) |
| **328** | ENSG00000164091 | TMEM113 | -0.84 | 7.17E-08 | WD repeat-containing protein 82;WDR82;ortholog | WD REPEAT-CONTAINING PROTEIN 82 (PTHR19861:SF0) |
| **329** | ENSG00000170471 | RALGAPB | -0.84 | 8.67E-08 | Ral GTPase-activating protein subunit beta;RALGAPB;ortholog | RAL GTPASE-ACTIVATING PROTEIN SUBUNIT BETA (PTHR21344:SF1) |
| **330** | ENSG00000187605 | TET3 | -0.84 | 6.94E-08 | Methylcytosine dioxygenase TET3;TET3;ortholog | METHYLCYTOSINE DIOXYGENASE TET3 (PTHR23358:SF4) |
| **331** | ENSG00000124422 | USP22 | -0.85 | 4.49E-08 | Ubiquitin carboxyl-terminal hydrolase 22;USP22;ortholog | UBIQUITIN CARBOXYL-TERMINAL HYDROLASE 22 (PTHR21646:SF40) |
| **332** | ENSG00000140396 | NCOA2 | -0.85 | 5.11E-08 | Nuclear receptor coactivator 2;NCOA2;ortholog | NUCLEAR RECEPTOR COACTIVATOR 2 (PTHR10684:SF2) |
| **333** | ENSG00000176994 | SMCR8 | -0.85 | 5.27E-08 | Guanine nucleotide exchange protein SMCR8;SMCR8;ortholog | GUANINE NUCLEOTIDE EXCHANGE PROTEIN SMCR8 (PTHR31334:SF1) |
| **334** | ENSG00000065526 | SPEN | -0.86 | 2.02E-08 | Msx2-interacting protein;SPEN;ortholog | MSX2-INTERACTING PROTEIN (PTHR23189:SF48) |
| **335** | ENSG00000099942 | CRKL | -0.86 | 2.26E-08 | Crk-like protein;CRKL;ortholog | CRK-LIKE PROTEIN (PTHR19969:SF20) |
| **336** | ENSG00000112624 | BICRAL | -0.86 | 2.34E-08 | BRD4-interacting chromatin-remodeling complex-associated protein-like;BICRAL;ortholog | BRD4-INTERACTING CHROMATIN-REMODELING COMPLEX-ASSOCIATED PROTEIN-LIKE (PTHR15572:SF2) |
| **337** | ENSG00000123066 | MED13L | -0.86 | 1.52E-08 | Mediator of RNA polymerase II transcription subunit 13-like;MED13L;ortholog | MEDIATOR OF RNA POLYMERASE II TRANSCRIPTION SUBUNIT 13-LIKE (PTHR48249:SF1) |
| **338** | ENSG00000156030 | C14orf43 | -0.86 | 1.66E-08 | Mitotic deacetylase-associated SANT domain protein;MIDEAS;ortholog | MITOTIC DEACETYLASE-ASSOCIATED SANT DOMAIN PROTEIN (PTHR16089:SF24) |
| **339** | ENSG00000198815 | FOXJ3 | -0.86 | 1.99E-08 | Forkhead box protein J3;FOXJ3;ortholog | FORKHEAD BOX PROTEIN J3 (PTHR46078:SF3) |
| **340** | ENSG00000253327 | RAD21-AS1 | -0.86 | 1.26E-08 |  |  |
| **341** | ENSG00000161021 | MAML1 | -0.87 | 7.62E-09 | Mastermind-like protein 1;MAML1;ortholog | MASTERMIND-LIKE PROTEIN 1 (PTHR15692:SF19) |
| **342** | ENSG00000163788 | SNRK | -0.87 | 7.26E-09 | SNF-related serine/threonine-protein kinase;SNRK;ortholog | SNF-RELATED SERINE/THREONINE-PROTEIN KINASE (PTHR24343:SF181) |
| **343** | ENSG00000171940 | ZNF217 | -0.87 | 9.89E-09 | Zinc finger protein 217;ZNF217;ortholog | ZINC FINGER PROTEIN 217 (PTHR45925:SF4) |
| **344** | ENSG00000110367 | DDX6 | -0.88 | 3.19E-09 | Probable ATP-dependent RNA helicase DDX6;DDX6;ortholog | ATP-DEPENDENT RNA HELICASE DDX6-RELATED (PTHR47960:SF2) |
| **345** | ENSG00000100811 | YY1 | -0.89 | 1.44E-09 | Transcriptional repressor protein YY1;YY1;ortholog | TRANSCRIPTIONAL REPRESSOR PROTEIN YY1 (PTHR14003:SF10) |
| **346** | ENSG00000140992 | PDPK1 | -0.89 | 1.55E-09 | 3-phosphoinositide-dependent protein kinase 1;PDPK1;ortholog | 3-PHOSPHOINOSITIDE-DEPENDENT PROTEIN KINASE 1-RELATED (PTHR24356:SF163) |
| **347** | ENSG00000197724 | PHF2 | -0.91 | 6.25E-11 | Lysine-specific demethylase PHF2;PHF2;ortholog | LYSINE-SPECIFIC DEMETHYLASE PHF2 (PTHR23123:SF14) |
| **348** | ENSG00000152518 | ZFP36L2 | -0.92 | 3.79E-11 | mRNA decay activator protein ZFP36L2;ZFP36L2;ortholog | MRNA DECAY ACTIVATOR PROTEIN ZFP36L2 (PTHR12547:SF156) |

## **Table S4: Phenotypic changes in twenty morbidly obese men at month 12 after Roux-en-Y gastric bypass**

|  | BASELINE | Post-RYGB (M12) | P-value |
| --- | --- | --- | --- |
| Body mass index (kg/m^2^) | 45.5 ± 8.3 | 32.3 ± 5.2 | **<0.0001** |
| Metabolic comorbidities |  |  |  |
| Metabolic syndrome | 15/20 | 4/20 | **0.003 #** |
| Arterial Hypertension | 14/20 | 6/20 | **0.03 #** |
| Diabetes or glucose intolerance | 16/20 | 5/20 | **0.003 #** |
| Dyslipidemia | 13/20 | 3/20 | **0.001 #** |
| Metabolic parameters |  |  |  |
| Glucose (mmol/l) | 6.1 ± 1.0 | 5.3 ± 0.4 | **0.0004** § |
| Insulin (mU/l) | 39 ± 26 | 12 ± 4.0 | **0.0007** § |
| HOMA-IR | 10.7 ± 8.2 | 2.7 ± 1.1 | **0.0007** § |
| Hb1Ac (%) | 6.3 ± 1.1 | 5.4 ± 0.4 | **0.0001** § |
| Total cholesterol (mmol/l) | 4.5 ± 0.8 | 4.0 ± 0.8 | **0.009** |
| Triglycerides (mmol/l) | 2.0 ± 1.1 | 1.0 ± 0.3 | **0.0002** § |
| HDL cholesterol (mmol/l) | 1.1 ± 0.3 | 1.5 ± 0.4 | **0.0001** § |
| ALAT (IU/l) | 42 ± 16 | 30 ± 15 | **0.02** § |
| ASAT (IU/l) | 29 ± 7 | 24 ± 6 | **0.008** |
| NAFLD Fibrosis Score | -0.36 ± 1.38 | -1.88 ± 0.99 | **<0.0001** |
| hsCRP (mg/l) | 7.2 ± 5.4 | 1.2 ± 1.2 | **0.0001** § |
| Leptin (ng/ml) | 64 ± 35 | 12 ± 9 | **0.0001** § |
| Fat mass percentage (%) | 45.8 ± 6.3 | 30.2 ± 8.1 | **<0.0001** |
| Visceral adipose tissue (g) | 4401 ± 1466 | 1344 ± 696 | **<0.0001** |
| Reproductive hormones |  |  |  |
| ADAM score, positive | 5/9 | 1/9 | 0.13 # |
| Total testosterone (nmol/l) | 9.6 ± 3.3 | 20.6 ± 6.8 | **< 0.0001** |
| SHBG (nmol/l) | 22 ± 9 | 43 ± 16 | **< 0.0001** |
| Calculated free testosterone (pmol/l) | 230 ± 72 | 368 ± 76 | **< 0.0001** |
| LH (IU/l) | 5.3 ± 2.5 | 6.6 ± 3.3 | **0.01** |
| FSH (IU/l) | 4.5 ± 3.5 | 4.9 ± 3.0 | 0.09 § |
| Estradiol (nmol/l) | 0.13 ± 0.05 | 0.12 ± 0.03 | 0.69 § |
| Testosterone/Estradiol ratio | 82 ± 35 | 182 ± 77 | **< 0.0001** |

All measurements were performed from fasting samples. RYGB, Roux-en-Y gastric bypass; Insulin levels and HOMA-IR of the two patients on exogenous insulin therapy were not included. Changes in all parameters were analyzed using ordinary paired t-test for parameters with normal distribution (otherwise, Wilcoxon test). § Non-normal distribution according to D'Agostino & Pearson test. **#,** McNemar’s test. Statistically significant differences (p<0.05) are shown in bold.

## **Table S5: Top ranked correlation relationships of clinical variables describing post-RYGB recovery with VAT gene expression**

| **Gene** | **Phenotypic variable** | **r** | **p value** | **Associated metabolic and reproductive traits (GWAS Catalog)** | **Relevant metabolic/reproductive functions** |
| --- | --- | --- | --- | --- | --- |
| *KHK* | Delta HOMA-IR | -0.94 | 7.21E-06 |  | Involved in fructose-induced metabolic syndrome in mice ^19^ |
| *CLIP3* | VAT percent decrease | -0.87 | 2.28E-05 |  |  |
| *C5orf34* | HOMA-IR percent decrease | 0.92 | 2.59E-05 |  | Marker of gestational diabetes ^20^ |
| *JPH4* | Delta Testosterone | -0.85 | 2.69E-05 |  |  |
| *CDSN* | VAT percent decrease | -0.86 | 3.87E-05 | Hip circumference adjusted for BMI | Marker of UCP1+ adipocytes in white adipose tissue (Brite adipocytes) ^21^ |
| *MIATNB* | Delta hs-CRP | 0.86 | 4.11E-05 |  |  |
| *CERS6* | Fat mass percent decrease | 0.86 | 4.15E-05 | Blood glucose levels Glycated hemoglobin levels Type 2 diabetes Triglyceride levels | Contributes to weight gain by promoting ceramide synthesis ^22^ |
| *PANK3* | VAT percent decrease | 0.86 | 4.71E-05 |  | Regulatory enzymes that control the rate of CoA biosynthesis and marker of white adipocytes ^23^. CoA plays a major role in mammalian physiology, particularly lipid utilization and energy production from lipid sources.^24^ |
| *SLC41A3* | Fat mass percent decrease | -0.85 | 4.94E-05 |  | Mediates mitochondrial Mg2+ efflux ^25^ |
| *SEL1L2* | Delta Testosterone | -0.84 | 4.96E-05 |  |  |
| *FAM72B* | Fat mass percent decrease | -0.84 | 7.48E-05 |  |  |
| *FLJ33814 (CCDC117)* | hs-CRP percent decrease | 0.84 | 7.88E-05 | Waist-to-hip ratio adjusted for BMI |  |
| *LRRC3* | VAT percent decrease | 0.84 | 9.41E-05 |  |  |
| *LINC02511* | Delta Testosterone | 0.82 | 9.42E-05 |  |  |
| *TOP3B* | Delta BMI | -0.82 | 1.09E-04 | Age at menopause |  |
| *FLVCR1* | Delta HOMA-IR | 0.89 | 1.18E-04 |  | Increased expression in adipose tissue of DT2 patients ^26^ |
| *WNT5B* | Delta VAT | -0.83 | 1.28E-04 |  | Regulator of adipogenesis and potential contributor of DT2 ^27^ |
| *UBOX5-AS1* | Delta Fat mass percent | -0.83 | 1.29E-04 |  |  |
| *CFL1P1* | Delta BMI | -0.81 | 1.33E-04 | Type 2 diabetes Waist-to-hip ratio adjusted for BMI Waist-hip ratio |  |
| *MPV17* | VAT percent decrease | -0.83 | 1.52E-04 | Triglyceride levels Sex hormone-binding globulin levels Total cholesterol levels C-reactive protein levels Apolipoprotein B levels |  |
| *CHAF1B* | Delta Testosterone | -0.81 | 1.53E-04 | Menarche (age at onset) |  |
| *TUSC1* | HOMA-IR percent decrease | -0.88 | 1.54E-04 |  |  |
| *AKAP10* | HOMA-IR percent decrease | 0.88 | 1.61E-04 | Insulin-like growth factor 1 levels |  |
| *CUL7* | Weight percent loss | -0.81 | 1.67E-04 |  | Modulate adipogenesis ^28^ |
| *HCN1* | Delta Testosterone | -0.80 | 1.76E-04 |  |  |
| *SLC41A3* | VAT percent decrease | -0.82 | 1.81E-04 |  | Mitochondrial Mg2+ efflux system ^25^ |
| *PFN4* | Fat mass percent decrease | -0.82 | 1.82E-04 |  |  |
| *ECT2* | Delta weight | 0.80 | 1.82E-04 | Triglyceride measurement High density lipoprotein cholesterol measurement |  |
| *CFL1P1* | Delta weight | -0.80 | 1.90E-04 | Type 2 diabetes Waist-to-hip ratio adjusted for BMI Waist-hip ratio |  |
| *CUL7* | BMI percent loss | -0.80 | 1.93E-04 |  | Regulator of adipogenesis ^28^ |
| *ACAT2* | HOMA-IR percent decrease | -0.87 | 1.97E-04 | Lipoprotein (a) levels Cholesteryl esters to total lipids ratio in medium LDL Phospholipids to total lipids ratio in medium LDL | Impairs adipocyte function by increasing cholesterol esterification ^29^ |
| *ANKAR* | HOMA-IR percent decrease | 0.87 | 2.01E-04 |  |  |
| *WDR38* | Delta BMI | 0.80 | 2.07E-04 | Waist-hip ratio |  |
| *ATP11C* | VAT percent decrease | 0.82 | 2.09E-04 |  | Mediates the transport of aminophospholipids ^30^ |
| *ADCY2* | Delta hs-CRP | -0.81 | 2.15E-04 |  | Adcy2 KO mice display higher circulating cholesterol levels  <https://www.mousephenotype.org/data/genes/MGI:99676> |
| *CHD4* | Delta Testosterone | -0.80 | 2.22E-04 | Triglycerides Type 2 diabetes | Involved in Adipocyte Thermogenesis ^31^ |
| *ATL1-gamma (BCL11B)* | Weight percent loss | 0.80 | 2.25E-04 | Total testosterone levels Body mass index Body weight | Regulator of adipogenesis ^32^ |
| *NRBP2* | Delta hs-CRP | -0.81 | 2.27E-04 |  |  |
| *ADPRH* | Delta Testosterone | 0.80 | 2.32E-04 |  |  |
| *DCAF12* | Delta hs-CRP | 0.81 | 2.39E-04 | GWAS DT2 - Testosterone |  |
| *APOBEC3B* | Delta Testosterone | -0.79 | 2.48E-04 |  |  |
| *NCAPD3* | Delta hs-CRP | -0.81 | 2.55E-04 |  |  |
| *TMEM63A* | Delta hs-CRP | -0.81 | 2.56E-04 |  |  |
| *MT-TQ* | Delta HOMA-IR | -0.86 | 2.85E-04 |  | Suggestively significant association with HOMA-B, HbA1c, and BMI ^33^ |
| *SMG1P1* | Delta hs-CRP | -0.81 | 2.88E-04 |  |  |
| *PCTK3 (CDK18)* | Delta Testosterone | -0.79 | 2.89E-04 |  | Enhanced in white adipose tissue after High Fat diet ^34^ |
| *MAP2K1* | Delta Testosterone | 0.79 | 3.07E-04 | Body mass index |  |
| *POLR3C* | Delta VAT | -0.80 | 3.46E-04 |  |  |
| *ZNF286B* | Delta Testosterone | -0.78 | 3.51E-04 |  |  |
| *RAPGEF3* | Delta hs-CRP | -0.80 | 3.60E-04 | Waist circumference adjusted for body mass index Waist-hip ratio Waist-to-hip ratio adjusted for BMI Body mass index |  |

## **Table S6: Comparison of characteristics of obese that consented vs declined protocol 2**

|  | Accepted Protocol 2 (n=21) | Declined Protocol 2 (n=11) | P-value |
| --- | --- | --- | --- |
| Age (years) | 44.1 ± 5.3 | 47.0 ± 11.9 | 0.15 § |
| Body mass index (kg/m^2^) | 46.0 ± 7.2 | 42.1 ± 7.6 | 0.10 § |
| Hypertension (treated) | 11/21 | 7/11 | 0.71 # |
| Hypercholesterolemia (treated) | 3/21 | 3/11 | 0.39 # |
| Diabetes or glucose intolerance | 10/21 | 6/11 | 0.99 # |
| Treatment for diabetes | 3/21 | 6/11 | **0.03** # |
| Metformin | 3/21 | 5/11 | 0.09 # |
| GLP1 agonist | 1/21 | 5/11 | **0.01** # |
| Other diabetes treatment | 0/21 | 4/11 | **0.009** # |
| CPAP for sleep apnea syndrome | 12/21 | 9/11 | 0.25 # |
| Total testosterone (nmol/l) | 11.6 ± 4.7 | 10.4 ± 3.2 | 0.45 |
| Fat mass percentage (%) | 47.8 ± 4.7 | 42.7 ± 6.0 | **0.01** |
| Visceral adipose tissue (g) | 4210 ± 1329 | 3816 ± 1540 | 0.47 |
| hsCRP (mg/l) | 7.3 ± 4.8 | 4.2 ± 3.7 | 0.08 |
| Glucose (mmol/l) | 6.1 ± 0.9 | 6.4 ± 1.5 | 0.88 § |
| Insulin (mU/l) | 37 ± 24 | 37 ± 23 | 0.99 |
| HOMA-IR | 10.1 ± 7.2 | 10.4 ± 7.7 | 0.93 |
| Hb1Ac (%) | 6.0 ± 0.8 | 6.5 ± 1.4 | 0.29 § |
| Triglycerides (mmol/l) | 1.7 ± 0.9 | 2.1 ± 1.1 | 0.27 § |
| HDL cholesterol (mmol/l) | 1.1 ± 0.2 | 1.1 ± 0.3 | 0.66 § |
| LDL cholesterol (mmol/l) | 2.6 ± 0.6 | 2.6 ± 0.9 | 0.83 |
| ALAT (U/l) | 43 ± 22 | 46 ± 14 | 0.43 § |

Quantitative parameters were analyzed with unpaired t-test or Mann-Whitney test in case of non-normal distribution according to Shapiro-Wilk test (marked with §; # Fisher’s exact test; other diabetes treatment included sitagliptine (n=2), empagliflozin (n=1) and insulin degludec (n=2); CPAP, continuous positive airway pressure; significant differences (p<0.05) are shown in bold.

#

# **REFERENCES**

1. Duvoisin C, Favre L, Allemann P, Fournier P, Demartines N, Suter M. Roux-en-Y Gastric Bypass: Ten-year Results in a Cohort of 658 Patients. *Ann Surg*. Dec 2018;268(6):1019-1025. doi:10.1097/SLA.0000000000002538

2. Beaulieu C, Allen PS. Determinants of anisotropic water diffusion in nerves. *Magn Reson Med*. Apr 1994;31(4):394-400. doi:10.1002/mrm.1910310408

3. Hagmann P, Jonasson L, Maeder P, Thiran JP, Wedeen VJ, Meuli R. Understanding diffusion MR imaging techniques: from scalar diffusion-weighted imaging to diffusion tensor imaging and beyond. *Radiographics*. Oct 2006;26 Suppl 1:S205-23. doi:10.1148/rg.26si065510

4. Grundy SM, Cleeman JI, Daniels SR, et al. Diagnosis and management of the metabolic syndrome: an American Heart Association/National Heart, Lung, and Blood Institute Scientific Statement. *Circulation*. Oct 25 2005;112(17):2735-52. doi:10.1161/CIRCULATIONAHA.105.169404

5. Angulo P, Hui JM, Marchesini G, et al. The NAFLD fibrosis score: a noninvasive system that identifies liver fibrosis in patients with NAFLD. *Hepatology*. Apr 2007;45(4):846-54. doi:10.1002/hep.21496

6. Vermeulen A, Verdonck L, Kaufman JM. A critical evaluation of simple methods for the estimation of free testosterone in serum. *J Clin Endocrinol Metab*. Oct 1999;84(10):3666-72. doi:10.1210/jcem.84.10.6079

7. Salamin O, Ponzetto F, Cauderay M, et al. Development and validation of an UHPLC-MS/MS method for extended serum steroid profiling in female populations. *Bioanalysis*. Jun 2020;12(11):753-768. doi:10.4155/bio-2020-0046

8. Ruell PA, Gass GC. Enzymatic measurement of 3-hydroxybutyrate in extracts of blood without neutralization. *Ann Clin Biochem*. Mar 1991;28 ( Pt 2):183-4. doi:10.1177/000456329102800211

9. Vistnes M, Christensen G, Omland T. Multiple cytokine biomarkers in heart failure. *Expert Review of Molecular Diagnostics*. 2014;10(2):147-157. doi:10.1586/erm.10.3

10. Checa A, Xu N, Sar DG, Haeggstrom JZ, Stahle M, Wheelock CE. Circulating levels of sphingosine-1-phosphate are elevated in severe, but not mild psoriasis and are unresponsive to anti-TNF-alpha treatment. *Sci Rep*. Jul 15 2015;5:12017. doi:10.1038/srep12017

11. Checa A, Khademi M, Sar DG, et al. Hexosylceramides as intrathecal markers of worsening disability in multiple sclerosis. *Mult Scler*. Sep 2015;21(10):1271-9. doi:10.1177/1352458514561908

12. Teav T, Gallart-Ayala H, van der Velpen V, Mehl F, Henry H, Ivanisevic J. Merged Targeted Quantification and Untargeted Profiling for Comprehensive Assessment of Acylcarnitine and Amino Acid Metabolism. *Anal Chem*. Sep 17 2019;91(18):11757-11769. doi:10.1021/acs.analchem.9b02373

13. Rossum GVDFL. *Python 3 : reference manual*. SohoBooks; 2009.

14. McKinney W. Data Structures for Statistical Computing in Python. presented at: Proceedings of the 9th Python in Science Conference; 2010;

15. Inc PT. Collaborative data science. Montréal, QC: Plotly Technologies Inc.; 2015.

16. Pedregosa F, Varoquaux G, Gramfort A, et al. Scikit-learn: Machine Learning in Python. *J Mach Learn Res*. Oct 2011;12:2825-2830.

17. Virtanen P, Gommers R, Oliphant TE, et al. SciPy 1.0: fundamental algorithms for scientific computing in Python (vol 33, pg 219, 2020). *Nat Methods*. Mar 2020;17(3):352-352. doi:10.1038/s41592-020-0772-5

18. Turpin-Nolan SM, Bruning JC. The role of ceramides in metabolic disorders: when size and localization matters. *Nat Rev Endocrinol*. Apr 2020;16(4):224-233. doi:10.1038/s41574-020-0320-5

19. Ishimoto T, Lanaspa MA, Le MT, et al. Opposing effects of fructokinase C and A isoforms on fructose-induced metabolic syndrome in mice. *Proc Natl Acad Sci U S A*. Mar 13 2012;109(11):4320-5. doi:10.1073/pnas.1119908109

20. Wang X, Huang J, Zheng Y, et al. Study on the relationship between DNA methylation of target CpG sites in peripheral blood and gestational diabetes during early pregnancy. *Sci Rep*. Oct 14 2021;11(1):20455. doi:10.1038/s41598-021-99836-2

21. Wang H, Liu L, Lin JZ, Aprahamian TR, Farmer SR. Browning of White Adipose Tissue with Roscovitine Induces a Distinct Population of UCP1(+) Adipocytes. *Cell Metab*. Dec 13 2016;24(6):835-847. doi:10.1016/j.cmet.2016.10.005

22. Turpin SM, Nicholls HT, Willmes DM, et al. Obesity-induced CerS6-dependent C16:0 ceramide production promotes weight gain and glucose intolerance. *Cell Metab*. Oct 7 2014;20(4):678-86. doi:10.1016/j.cmet.2014.08.002

23. Robishaw JD, Neely JR. Coenzyme A metabolism. *Am J Physiol*. Jan 1985;248(1 Pt 1):E1-9. doi:10.1152/ajpendo.1985.248.1.E1

24. Dansie LE, Reeves S, Miller K, et al. Physiological roles of the pantothenate kinases. *Biochem Soc Trans*. Aug 2014;42(4):1033-6. doi:10.1042/BST20140096

25. Mastrototaro L, Smorodchenko A, Aschenbach JR, Kolisek M, Sponder G. Solute carrier 41A3 encodes for a mitochondrial Mg(2+) efflux system. *Sci Rep*. Jun 15 2016;6:27999. doi:10.1038/srep27999

26. Moreno-Navarrete JM, Rodriguez A, Ortega F, et al. Increased adipose tissue heme levels and exportation are associated with altered systemic glucose metabolism. *Sci Rep*. Jul 13 2017;7(1):5305. doi:10.1038/s41598-017-05597-2

27. Kanazawa A, Tsukada S, Sekine A, et al. Association of the gene encoding wingless-type mammary tumor virus integration-site family member 5B (WNT5B) with type 2 diabetes. *Am J Hum Genet*. Nov 2004;75(5):832-43. doi:10.1086/425340

28. Zhang W, Wang Q, Song P, Zou MH. Liver kinase b1 is required for white adipose tissue growth and differentiation. *Diabetes*. Jul 2013;62(7):2347-58. doi:10.2337/db12-1229

29. Xu Y, Du X, Turner N, Brown AJ, Yang H. Enhanced acyl-CoA:cholesterol acyltransferase activity increases cholesterol levels on the lipid droplet surface and impairs adipocyte function. *J Biol Chem*. Dec 13 2019;294(50):19306-19321. doi:10.1074/jbc.RA119.011160

30. Shin HW, Takatsu H. Substrates of P4-ATPases: beyond aminophospholipids (phosphatidylserine and phosphatidylethanolamine). *FASEB J*. Mar 2019;33(3):3087-3096. doi:10.1096/fj.201801873R

31. Nie T, Hui X, Mao L, et al. Harmine Induces Adipocyte Thermogenesis through RAC1-MEK-ERK-CHD4 Axis. *Sci Rep*. Nov 2 2016;6:36382. doi:10.1038/srep36382

32. Inoue J, Ihara Y, Tsukamoto D, et al. Identification of BCL11B as a regulator of adipogenesis. *Sci Rep*. Sep 2 2016;6:32750. doi:10.1038/srep32750

33. Kraja AT, Liu C, Fetterman JL, et al. Associations of Mitochondrial and Nuclear Mitochondrial Variants and Genes with Seven Metabolic Traits. *Am J Hum Genet*. Jan 3 2019;104(1):112-138. doi:10.1016/j.ajhg.2018.12.001

34. Shaik AA, Qiu B, Wee S, Choi H, Gunaratne J, Tergaonkar V. Phosphoprotein network analysis of white adipose tissues unveils deregulated pathways in response to high-fat diet. *Sci Rep*. May 16 2016;6:25844. doi:10.1038/srep25844
